# The impact of asymmetrical gene expression on the development of spike morphology in *Triticum aestivum*

**DOI:** 10.1002/imo2.70047

**Published:** 2025-08-27

**Authors:** Fang He, Xiaojuan Liu, Qian Ma, Wei Wan, Luhua Li, Kuiyin Li, Zhenzhen Jia, Suqin Zhang, Ruhong Xu, Mingjian Ren

**Affiliations:** ^1^ Guizhou Subcenter of National Wheat Improvement Center, Key Laboratory of Functional Agriculture of Guizhou Provincial Higher Education Institutions Guizhou University Guiyang China; ^2^ Anshun University Anshun China; ^3^ School of Life Sciences Guizhou Normal University Guiyang China

**Keywords:** asymmetrical gene expression, single cell, transcriptome, wheat spike

## Abstract

Wheat yield is primarily determined by panicle density per unit area, grain count per spike, and grain weight. The proliferation of wheat spikes affects both the number of grains per spike and grain weight. However, the molecular regulatory mechanisms of wheat spike development are still largely elusive. In this study, we acquired high‐quality sequencing data from 5989 cells derived from the double‐ridge stage spike of a common wheat variety Jimai 22. The data revealed the presence of 10 distinct cell types, which were validated using RNA in situ hybridization and cell type‐specific gene expression. The transition from promeristem to protoxylem and protophloem cells signifies the initiation of differentiation for protoxylem and primary protophloem cells within the promeristem. This process results in five distinct cellular differentiation states that correspond to the expression of 1410 genes. In wheat spikes, differential gene expression across eight developmental stages revealed seven unique expression patterns. Specifically, genes differentially expressed in stages C3, C4, C5, and C7 were identified as being uniquely active during the anther meristem, double ridge, floral meristem, and pistil primordium stages, respectively. Furthermore, the differential genes in stage C2 are likely to encompass critical genes that regulate the reproductive growth of wheat spikes, while those in stage C1 may significantly influence floret creation and development. Additionally, the transition of gene triplets between suppressed and balanced types represents a key element affecting spike differentiation. In this context, dominant gene triplets primarily fulfill functions associated with housekeeping genes. This study explores the impact of asymmetrical gene triplet expression during spike development on the regulation of wheat yield traits, utilizing the single‐cell transcriptome atlas of the wheat spike. Our analysis of homologous gene asymmetrical expression throughout development, coupled with single‐cell resolution, suggests this asymmetry could be a pivotal factor in cell differentiation.

## Highlights

- We constructed the first single‐cell transcriptome atlas of wheat spike, identifying 10 distinct cell types.
- Pseudotime trajectory analysis revealed protoxylem and protophloem differentiation originating from promeristem cells.
- Gene triplet transitions from suppressed to balanced types regulate wheat spike differentiation.
- Subgenome expression asymmetry influences cell differentiation dynamics during spike development.

## Graphical abstract

The schematic illustration depicting the production process of wheat spikes and spikelets demonstrates the morphological traits at each developmental stage. The double ridge (DR) phase single‐cell transcriptome analysis, in conjunction with RNA in situ hybridization, categorized 13 clusters into 10 distinct cell types. Analysis of homologous gene dynamics revealed that dominant‐type triads were highly conserved during wheat spike development. Frequent transitions occurred between suppressed and balanced‐type triads: suppressed‐to‐balanced transitions predominated from elongation stage (EL) to inflorescence meristem stage (IM), IM to DR, glume primordium differentiation stage (GP) to floral meristem stage (FM), and pistil primordium stage (PP) to anther meristem stage (AM), while balanced‐to‐suppressed transitions prevailed from DR to GP, GP to FM and FM to PP (C).

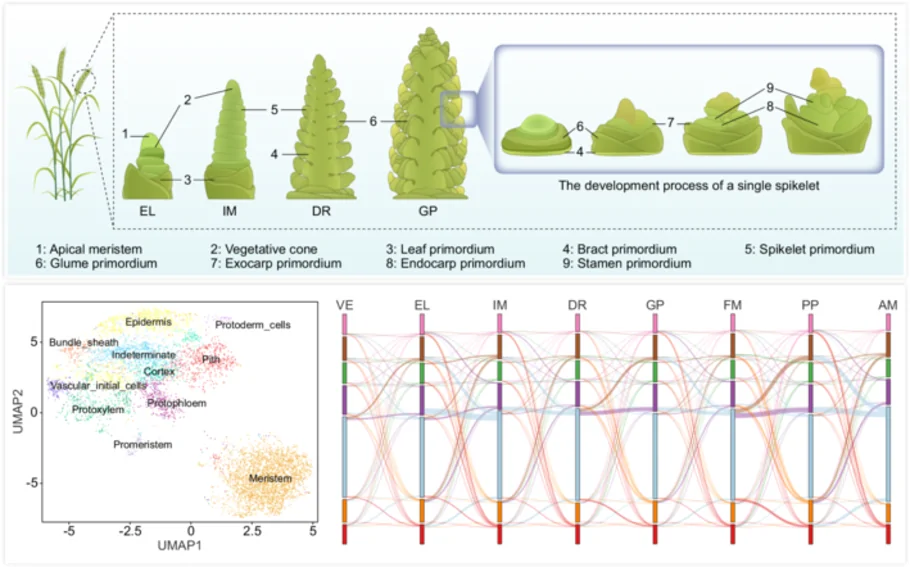

## 1 INTRODUCTION

Allopolyploidization is a critical biological mechanism that significantly contributes to plant speciation and evolution, arguably surpassing other evolutionary processes in the development of vascular plants [1, 2, 3, 4]. This process leads to the formation of hybrid species that contain two or more distinct genomes within a single nucleus. Consequently, newly established allopolyploid species must achieve exclusive intra‐genomic pairing during meiosis to ensure full fertility and disomic inheritance, which necessitates cytological diploidization [5]. Cytological diploidization supports two opposing yet complementary phenomena that enhance the evolutionary success of allopolyploids: (i) the accumulation and preservation of stable and advantageous inter‐genomic genetic combinations, facilitated by the absence of homoeologous pairing and recombination, and (ii) genomic asymmetry that influences various morphological, physiological, and molecular traits, characterized by the exclusive or predominant influence of one constituent genome on specific traits [6, 7, 8]. *Triticum aestivum* (2*n* = 6× = 42, AABBDD) originated through two instances of polyploidization. Its meiotic stability and the preservation of homologous gene order make it a valuable species for investigating the effects of recent polyploidy on gene expression [9, 10]. Research on the transcriptome of common wheat has revealed frequent stage‐specific transcriptional expression and subgenomic asymmetry [8, 11, 12, 13]. Furthermore, it has been observed that this expression asymmetry may impact phenotypic outcomes [14]. However, current understanding of the asymmetrical expression of homologous genes in common wheat is largely based on data derived from specific tissues or organs, which consist of a mixture of various cell types. Consequently, the exact mechanisms underlying asymmetric expression in heterogeneous cells remain poorly understood, primarily due to the lack of reference single‐cell transcriptional patterns.

Plant cells serve as the fundamental building blocks for growth and development in plants. Each cell type fulfills a distinct biological role that contributes to plant adaptation and overall development in response to environmental conditions [15, 16, 17, 18]. Single‐cell transcriptome sequencing (scRNA‐seq) facilitates the mapping and quantification of transcriptional activity at the individual cell level. This technique enables the efficient identification of rare and novel cell types, as well as the simultaneous characterization of multiple cell types and states, thereby providing a more precise and comprehensive understanding of their roles in various life processes [19, 20]. ScRNA‐seq has been extensively employed in animal studies, and it has recently gained traction in plant research, particularly for identifying cell identity [21, 22], constructing expression trajectories [23, 24, 25], and unraveling gene regulatory networks [26]. Previous studies on single‐cell analysis in plants have highlighted the significance and potential of scRNA‐seq technology within the field. Wheat, a major cereal crop globally, has spike features that are crucial for determining yield. Current knowledge regarding spike and floral organ development in wheat predominantly focuses on tissue variability. However, it is increasingly evident that heterogeneity exists among different cells within the same tissue, particularly during the critical phase of plant stem cell differentiation. Therefore, elucidating the cell type composition of wheat spikes and the developmental changes of each cell type during the establishment of spike morphology is of great importance for advancing wheat growth and development research.

All the above‐ground portions of plants are derived from the apical meristem (SAM) located at the tip of the stem. The processes of initiating, maintaining, and transforming the SAM into the inflorescence meristem (IM) are critical for defining the plant's overall shape and productivity. In wheat, the arrangement of spikes within the inflorescence is limited, with each spike connected to a node on the rachis and containing several florets at each node. The maximum number of seeds per spike is influenced by both the total number of spikes and the number of florets present on each spike. Therefore, the development of spikes is a critical biological process affecting grain number and yield in wheat. This developmental process consists of 10 distinct stages, each characterized by unique attributes and significance [27, 28]. The changes occurring during these stages are generally linked to the quantity of spikes, their spatial arrangement, and the fertility of the florets. Key genes regulating the structure of flower clusters have been reported in cereal crops such as rice, maize (*Zea mays*), and barley [29, 30, 31, 32]. Currently, the predominant strategies for identifying genes associated with wheat spike development involve homologous cloning, which compares these genes with known counterparts in *Arabidopsis thaliana* and rice, as well as map‐based cloning utilizing linkage mapping or genome‐wide association studies [33, 34, 35]. Furthermore, targeting induced local lesions in genomes mutants play a significant role in identifying genes related to spike morphology and their respective functions [36]. The ongoing updates to the wheat reference genome [37, 38, 39, 40], coupled with the accumulation of extensive germplasm resources [41, 42], the establishment of mutant libraries [43, 44], the development of high‐throughput phenotyping platforms [45, 46], and advancements in genome editing tools [47, 48, 49], alongside improvements in wheat genetic transformation efficiency, are poised to expedite research aimed at isolating genes linked to spike morphogenesis. Notably, the recent development and application of single‐cell sequencing technology have greatly enhanced our ability to investigate the morphogenesis and gene regulation of wheat spikes at the cellular level [50, 51, 52].

*T. aestivum* is a crucial global staple crop, and enhancing wheat production primarily relies on increasing the number of wheat spikes and the weight of a thousand grains. The development of wheat spikes is a critical phase in the growth cycle, significantly influencing the grain count per spike and the thousand‐grain weight at harvest. In this study, we conducted a single‐cell transcriptome analysis of wheat spikes at the double ridge stage (DR), together with transcriptome data from the vegetative stage (VE) to the anther meristem stage (AM). We have created a single‐cell transcriptome atlas that captures the developmental patterns of protoxylem and protophloem cells within the wheat spike. Furthermore, we explored gene expression changes during the development of wheat spikes at the individual cell level, with a particular focus on asymmetrical expression patterns. Our objective is to provide valuable resources regarding the regulation of gene expression in wheat spike development, the evolution of various cell types, and the identification of specific cellular features that are significant for agricultural advancement.

## 2 RESULTS

### Single‐cell transcriptome atlas of wheat spike

Following the vernalization of greenhouse‐grown JM22 at a low temperature, the spikes at the double ridge stage (DR_JM) were identified through microscopic examination in the enzymatic solution for protoplast preparation. The protoplast suspension was analyzed for cell counting and activity test, revealing a concentration of 510 cells/μL, a total cell count of 35,700, and a cell viability of 92%. It meets the criteria for scRNA‐seq.

**FIGURE 1:**
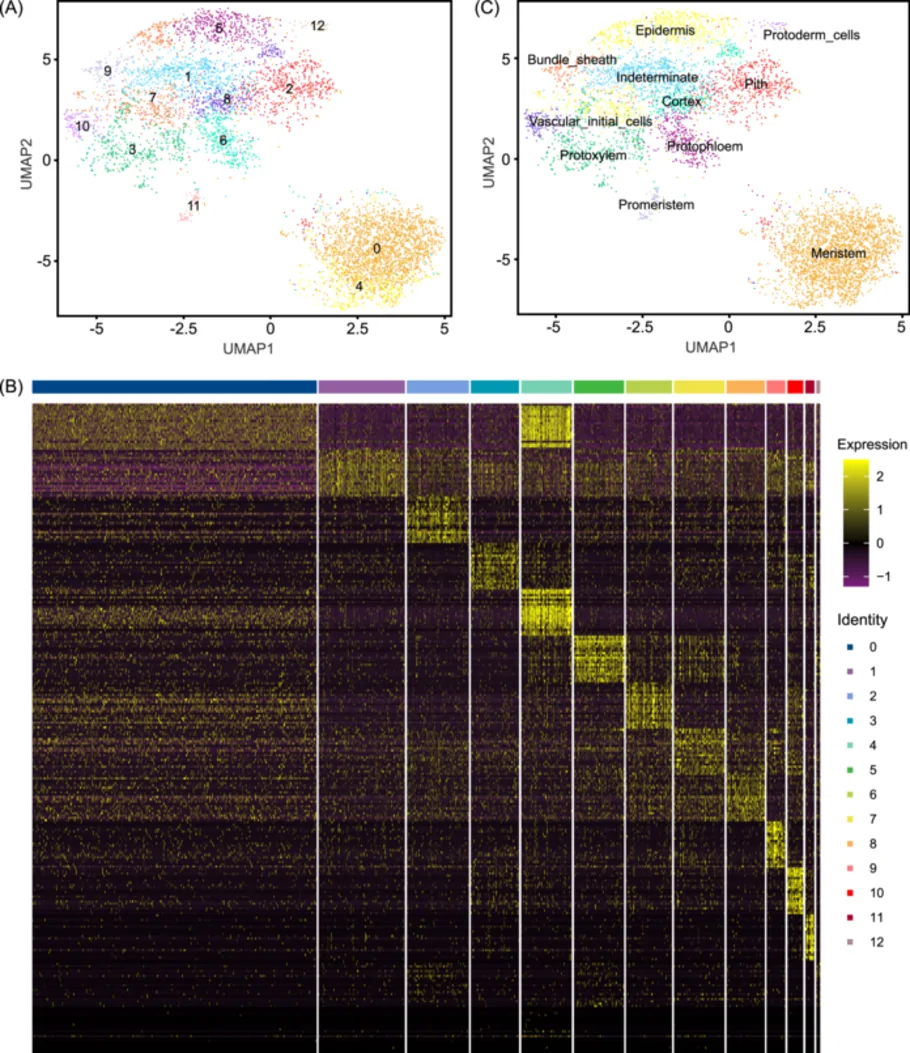
Single‐cell transcriptome analysis of wheat spikes. (A) Single‐cell cluster clustering UMAP plot, classification of all cells into 13 clusters. (B) Top 20 gene‐specific expression patterns in each cluster. (C) UMAP plot of wheat spike cell types, identification of 13 clusters into 11 cell types. UMAP, Uniform Manifold Approximation and Projection.

The DR_JM samples were subjected to sequencing, resulting in a total of 371,653,049 reads. After filtering low‐quality reads, the clean reads were then mapped to the reference genome, resulting a mapping rate of 88.9% (Figure S1). Following the application of CellRanger for quality control of sequencing data, we successfully obtained a final expression matrix consisting of 6564 cells. In this matrix, we detected a total of 87,695 genes. Overall, around 97.5% of reads had the correct barcode on average, 5182 unique molecular identifiers (UMIs) were found in each cell (Table S1). The quality control (QC) results showed that the wheat scRNA‐seq data produced in this investigation was comparable to the published single‐cell data set of common wheat Aikang 58 (AK58) root tips (Table S1) [51]. The cell‐gene expression matrices were imported into DoubletFinder (v.2.0.3) and Seurat (v.3.1.0) for processing. This resulted in a total of 5989 good‐quality cells with an average of 2731 UMIs per cell (Table S1). In the end, our unsupervised technique categorized the cells into 13 distinct cellular clusters (Figure 1A, Figures S2, and S3A,B). Among these clusters, cluster 0 has the largest number of cells (Table S2).

Significant resemblance of gene expression was observed between cluster 0 and cluster 4 with a high correlation (*r* = 0.74) and similar gene expression patterns (Figure S3C), suggesting that they may belong to the same cell type and perform similar tasks. Conversely, the correlation between cluster 4 and the other cellular cluster was low. Regarding the remaining cell clusters, while some clusters exhibit more apparent resemblance, there are still discernible distinctions in the roles they perform. This indicates the existence of subdivisions within these cell clusters. To identify the cell types corresponding to the cell groups obtained, we utilized Seurat's rank sum test. This statistical method enabled us to analyze the differential expression of genes within each individual cell group, thereby allowing for the identification of genes that were upregulated in each category. The results revealed that the distribution of the top 20 upregulated genes in the DR_JM group exhibited a diagonal pattern, which suggests that our classification of the clusters for the two materials was accurate (Figure 1B).

### Annotation of cell types in wheat spike

To identify cell types within single‐cell transcriptome data, we initially compiled a list of RNA in situ hybridization‐validated cell type marker genes from Arabidopsis, rice, and maize. Utilizing the homologous genes corresponding to these marker genes in wheat, we conducted a screening for their specific expression within various clusters. This analysis confirmed that cluster 0 and cluster 4 are associated with the meristem, cluster 3 corresponds to the protoxylem, and cluster 11 is linked to the promeristem. Subsequently, we employed the homologous genes of differentially expressed genes (DEGs) that are highly expressed in each cluster from Arabidopsis, rice, and maize. By examining the specific expression of these homologous genes across distinct cell types, we established that cluster 5 is representative of the epidermis, cluster 6 corresponds to the protophloem, and cluster 9 is associated with the bundle sheath (Figure 1C).

*Zm00001d047787* was confirmed to exhibit high expression levels in the meristem of the maize ear, whereas *Zm00001d045445* and *Zm00001d026129* demonstrated elevated expression levels in the promeristem of the ear and were classified as G2‐M dividing cells [53, 54, 55]. The homologs of *Zm00001d047787* in wheat, namely *TraesCS4D02G205000*, *TraesCS4A02G100100*, and *TraesCS4B02G204200*, are categorized as DEGs within cluster 4. Moreover, the gene *TraesCS5D02G110600*, which serves as a marker for the root meristem in wheat, showed significant expression in cluster 4. Consequently, it was initially inferred that cluster 4 likely corresponds to the meristem. Uniform Manifold Approximation and Projection (UMAP) clustering analysis indicated a greater overlap between the cells in cluster 0 and cluster 4, suggesting that cluster 0 may also be associated with meristematic activity. The genes *TraesCS7D02G068600* and *TraesCS2D02G401700*, which are homologous to *Zm00001d045445* and *Zm00001d026129*, respectively, exhibited high expression levels in cluster 11. Additionally, *TraesCS2D02G401700* and *TraesCS5D02G110600*, recognized as a marker gene for meristem in wheat roots, were both highly expressed in cluster 11 together with other meristematic cell marker genes *TraesCS2A02G443400*, *TraesCS2D02G442600*, and *TraesCS3A02G186500*. Therefore, cluster 11 is believed to be part of the promeristem. Similarly, the homologous genes of *Zm00001d005740* [56], *TraesCS5B02G229000*, and *TraesCS5D02G237300*, which have been validated through RNA in situ hybridization, along with the gene *TraesCS5A02G230500*, identified as a marker for protoxylem in wheat roots, demonstrated high expression levels in cluster 3. This observation implies that cluster 3 is representative of protoxylem (Figure 1C). Additionally, the DEGs *TraesCS7D02G444200* and *TraesCS7B02G355700* in cluster 5, showed sequence similarities to *CYP77B1* (*AT1G11600*) in Arabidopsis and *CYP77A3* (*Zm00001d014459*) in maize. *CYP77B1* is involved in polyhydroxy fatty acid biosynthesis in Arabidopsis [57], is associated with cuticle (wax) deposition, and exhibits abundant expression in extensive cellulose deposition cells, according to previous investigations [58, 59]. *CYP77A3* is a member of the CYP77 subfamily within the cytochrome P450 superfamily. It primarily facilitates fatty acid hydroxylation processes and contributes to the formation of the plant cuticle [60]. These two genes act as markers for the epidermis, suggesting that cluster 5 is associated with epidermal tissue. The 13 cell groups identified through UMAP clustering were systematically organized from cluster 0 to cluster 12, comprising the following: meristem, indeterminate, pith, protoxylem, meristem, epidermis, protophloem, epidermis, cortex, bundle sheath, vascular initial cells, promeristem, and protoderm cells. The apical meristem is constituted by the promeristem and meristem, while the procambium includes both the protoxylem and protophloem. Additionally, the ground meristem is formed from the pith and cortex (Figure 1C).

The marker genes specific to each cell type were validated through RNA in situ hybridization (Table S3). The results demonstrated that the gene *TraesCS6A02G063900* is specifically expressed in cortex cells, *TraesCS4D02G205000* is found in meristem tissues, *TraesCS2A02G404800* is present in promeristem tissues, and *TraesCS4A02G392300* is localized in vascular bundle sheaths (Figure S4). These findings are consistent with the results of cell typing, thereby reinforcing the reliability of the cytotyping outcomes.

### Trajectories of protophylem and promeristem tissues in wheat spike

**FIGURE 2:**
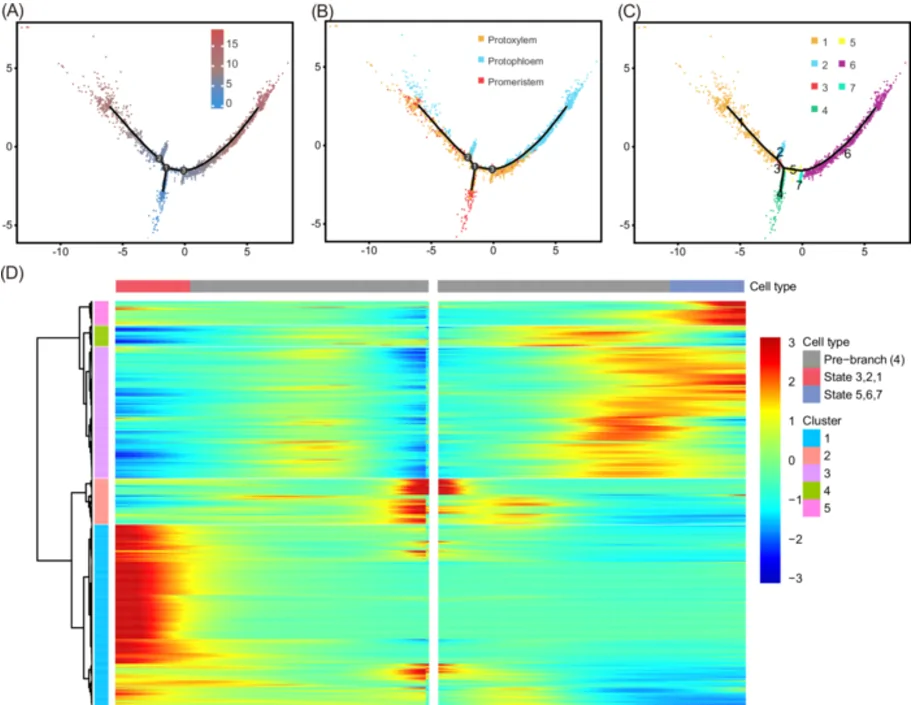
Trajectories of protoxylem and protophloem cell differentiation in wheat spike. (A) Pseudo‐time data of protoxylem and protophloem cells visualized using Monocle2. The color coding of data points (0−15) represents the continuous characteristics of cell differentiation. The pseudo‐time trajectory has a parabolic form, signifying the ongoing progression of cell states along a specified path. The three nodes marked on the trajectory are posited to signify pivotal turning moments, with node 1 potentially instigating the bifurcation of cell differentiation, whereas nodes 2 and 3 may be associated with distinct functional states. This indicates the existence of stage‐specific regulatory mechanisms in the differentiation of wheat shoot apical cells. (B) Cell types of differentiation trajectories designated by Monocle2. The three cell types—protoxylem (yellow), protophloem (light blue), and promeristem (red)—emonstrate unique spatial clustering properties in the trajectory map. The pseudo‐time trajectory, depicted by the black curve, delineates the possible pathways of cellular differentiation from promeristem to protoxylem and protophloem. (C) Differentiation status of protoxylem and protophloem cells. Colored points denote various differentiation states, while the thick black line illustrates the course of cell differentiation, representing the dynamic changes occurring throughout this process. There are seven unique states of cell differentiation: state 4 is located in the initial stage, whilst states 1 and 6 are at the end stages. States 2, 3, 5, and 7 are situated in the transitional stages of differentiation. (D) Expression heatmap of 1410 significantly differentially expressed genes (DEGs) with assigned temporal values, where each row corresponds to a gene clustered into five distinct expression patterns. The vertical and horizontal coordinates of A−C are both components. DEGs, differentially expressed genes.

This study conducted a trajectory analysis of the cells in the promeristem, protoxylem, and protophloem of early wheat spikes using Monocle 2. The investigation revealed that these cells ultimately specialized into two distinct states as they advanced along their developmental pathway, beginning from clusters of blue cells (Figure 2A). Moreover, individual cells from both time points exhibited a remarkably consistent temporal order, suggesting that the developmental trajectory during spike development is conserved. Marker gene tagging of cell types indicated that the developmental trajectory commenced in the promeristem and culminated in the two branches of the protophloem and protoxylem, respectively, following the point of divergence (Figure 2B). Upon assessing the differentiation states of these cells, it was found that the cells in the promeristem, protoxylem, and protophloem exhibited seven distinct differentiation states, labeled as states 1 to 7. State 4 was predominantly observed in the promeristem branches, while states 5 to 7 were primarily present in the protophloem branches. States 1 to 3 were mainly located in the protophloem branches (Figure 2B,C).

A total of 1410 genes exhibited significantly different expression levels in the pseudo‐time analysis. A heat map was generated to visualize the expression patterns of these genes in relation to their temporal values. Genes displaying similar expression trends during development were categorized into five distinct clusters (Figure 2D). Clusters 1 and 5 were expressed sequentially along the trajectory from the promeristem to the protoxylem and protophloem, respectively. This indicates that the genes in cluster 1 may facilitate the differentiation of promeristem cells into protoxylem, while the genes in cluster 5 may promote the differentiation of promeristem cells into protophloem. Conversely, the genes in cluster 2 exhibited high expression levels in the promeristem, with their expression declining following differentiation into protoxylem and protophloem. This observation suggests that the genes in cluster 2 may be involved in sustaining the biological functions of the promeristem. In contrast, clusters 3 and 4 comprise genes that are highly expressed in both the promeristem and protophloem before the acquisition of their final biological functions. This implies that these genes may play a critical role in the final morphological development of the protoxylem and protophloem. The Gene Ontology (GO) enrichment analysis of the DEGs revealed significant enrichment in 21 biological processes, including metabolic, developmental, and signaling activities. At the molecular function level, these genes were predominantly linked to 14 functional categories, encompassing binding, catalytic activity, and nucleic acid binding transcription factor activity. The genes were primarily situated within organelles and membrane systems (Figure S5).

### Transcriptome analysis of wheat during spike development

**FIGURE 3:**
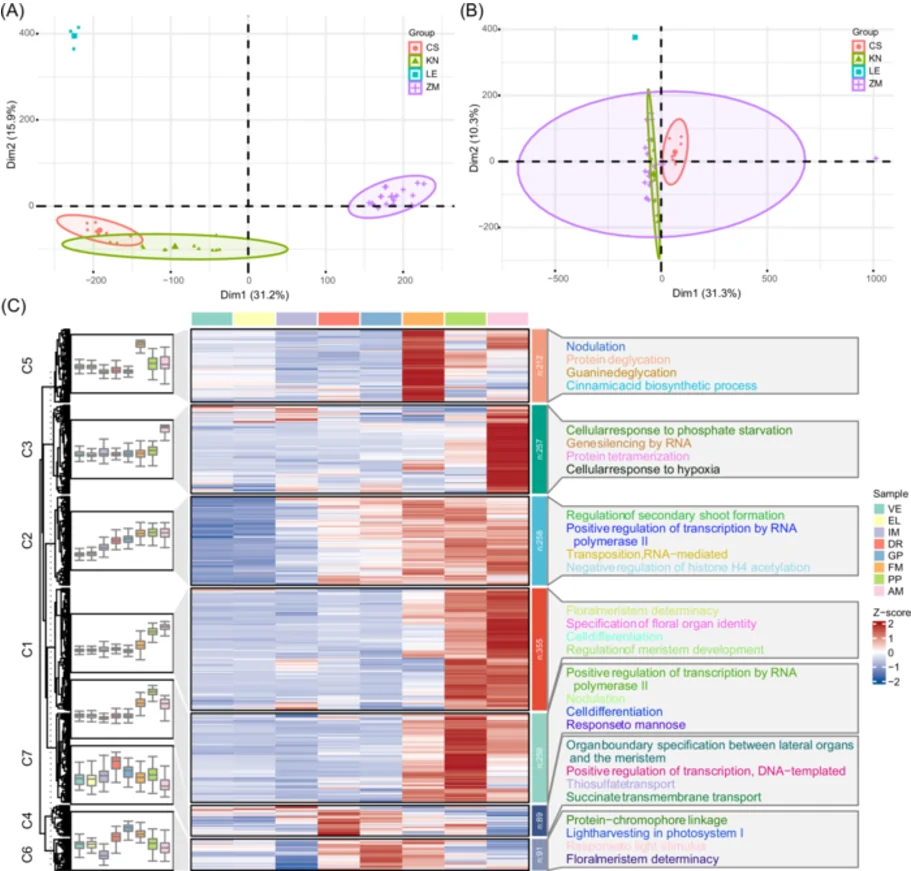
Transcriptome analysis of wheat spike. (A) Principal component analysis (PCA) analysis of the original data, the samples were aggregated separately with their respective sources. (B) PCA analysis of the transcriptome data after removing the batch effect, wheat spike samples were aggregated together, and samples from leaves were aggregated separately. (C) K‐means clustering of differentially expressed genes (DEGs) during the development of wheat spikes, expression trends of DEGs on the left, expression of the DEGs at each developmental period in the center, and GO enrichment for each cluster on the right. AM, anther meristem stage; DR, double ridge; EL, elongation stage; FM, floral meristem stage; GP, glume primordium differentiation stage; IM, inflorescence meristem stage; PP, pistil primordium stage; VE, vegetative stage.

We analyzed the raw transcriptome sequencing data (Table S4) across eight developmental stages of spike formation in wheat. The data were categorized based on their source and species, which revealed significant batch effects (Figure 3A). All samples, with the exception of the leaf transcriptome, clustered together after these bulk effects were eliminated (Figure 3B), suggesting the processed transcriptome is suitable for further analysis.

Genome‐wide differential expression studies were conducted to investigate the variety and potential functional disparities among distinct developmental phases of the wheat spike. A total of 1521 genes exhibiting differential expression between development phases were identified using the edgeR program (Figure S6). The changes in DEGs across eight stages of wheat spike development were further analyzed using k‐means clustering. The results revealed that the DEGs could be categorized into seven distinct clusters, with genes within each cluster displaying similar expression patterns (C1–C7). Notably, expression of genes in cluster C4 was significantly higher at DR compared to other stages. In contrast, genes cluster C5 were predominantly expressed at the floral meristem stage (FM). The differential genes within these two clusters were implicated in the differentiation of organ boundaries between lateral organs and meristem tissues, as well as in the processes of positive regulation of transcription, protein synthesis, guanine degradation, and cinnamic acid biosynthesis, as indicated by the GO enrichment analysis. The differential genes in C3 exhibited elevated expression exclusively during the AM. GO analysis revealed significant enrichment in cellular responses to phosphorus deprivation, RNA‐mediated gene silencing, protein tetramerization, and cellular responses to hypoxia. In contrast, the DEGs in C1 exhibited elevated expression levels during the FM, ultimately reaching their peak during the AM stage. These genes were shown to be enriched for the functions related to the determination of floral organ characteristics, cell differentiation, and regulation of meristem development. Simultaneously, the genes demonstrating differential expression in C2 were consistently upregulated during the FM and IM. These genes were enriched for the regulation of secondary shoot formation, positive regulation of transcription by RNA polymerase II, transposition, and negatively regulated GO biological processes by histone H4 acetylation. The expression levels of DEGs in C6 commenced to decrease during the VE and elongation stage (EL) stages, subsequently increased after the IM stage, and ultimately culminated during the glume primordium differentiation stage (GP) stage (Figure 3C). The results indicate that the genes expressed in C3, C4, C5, and C7 are specifically related to the AM, DR, FM, and pistil primordium stage (PP), respectively. In addition, the genes expressed in C2 are involved throughout the transition from nutritive growth to reproductive growth in the wheat spike and may include key regulators of reproductive growth in these spikes. Conversely, the genes expressed in C1 primarily regulate the reproductive growth of the wheat spike, with the DEGs in C1 predominantly participating in the morphogenesis and maturation of the wheat spike following FM, potentially exerting a critical influence on the formation and development of florets.

To evaluate the precision of the transcriptome data, we selected 54 genes that exhibited relatively high mRNA levels (log_2_FoldChange > 1) during both DR and FM. Specific primers for these genes, detailed in Table S5, were designed and their expression was confirmed using qPCR on wheat spike samples. The expression patterns of these genes were analyzed across six stages of wheat spike development: EL, IM, DR, GP, FM, and PP. The gene expression patterns observed during these six stages of spike formation revealed that the selected genes showed consistent quantitative changes in both qPCR and transcriptome analyses (Figure S7A–I). Additionally, the expression patterns demonstrated a strong correlation with the transcriptome data (*r*² = 0.819), indicating that the findings from the transcriptome analysis conducted in this study are highly reliable (Figure S7J).

### Weighted gene co‐expression network analysis of the wheat spike developmental phase

**FIGURE 4:**
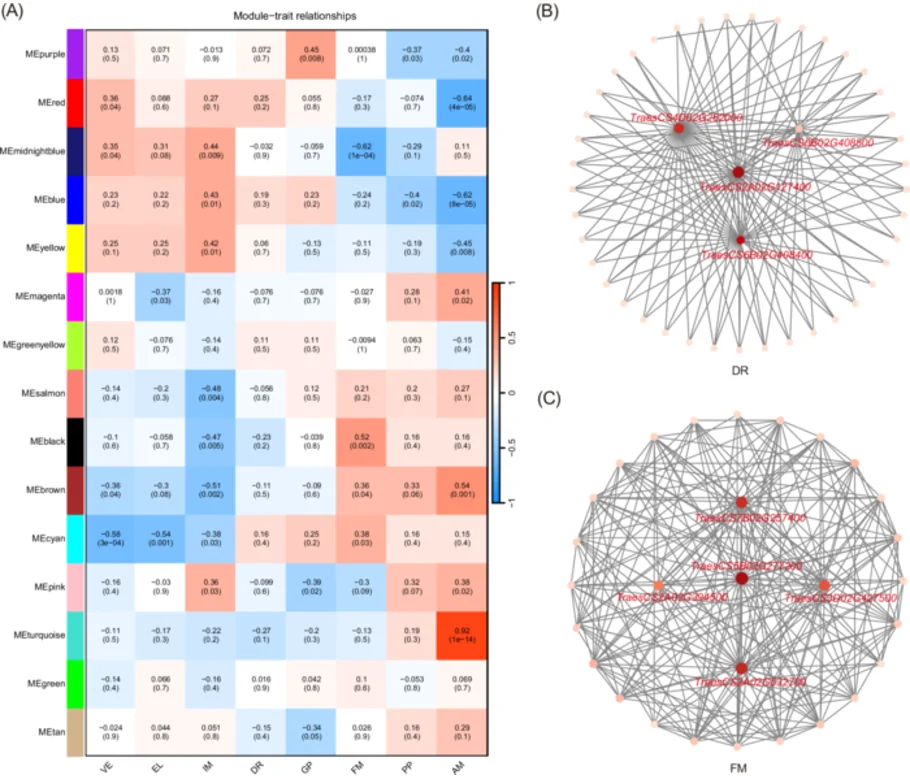
Heatmap illustrating the link between the developmental period and the identification of hub genes for wheat spike development. (A) Heat map illustrating the association between the module and the development of the wheat spike. Every column denotes the co‐expression module. The red hue of each box signifies the positive association between module and cell types. The blue hue of each box signifies the adverse associations between module and cell types. The numbers in the rectangular boxes represent the correlation coefficients and corresponding *p* values between the modules and the periods. DR, double ridge. (B) Co‐expression network of eight genes within the MEred module. (C) Co‐expression network of 117 genes within the MEblack module. FM, floral meristem stage. The dots darker hues and larger volumes signify greater weights.

To elucidate the principal genes implicated in wheat spike formation, we performed a weighted gene co‐expression network analysis on all expressed genes during the wheat spike growth process. Through cluster analysis of the correlation coefficients of expression levels across diverse samples (Figure S8A,B), we eliminated samples with low correlation (DR_KN_R2 and PP_ZM_R3), yielding a subset of samples that had strong correlation (Figure S8B). We determined the weight values for the filtered samples utilizing the pick Soft Threshold function from the weighted correlation network analysis (WGCNA) package (Figure S8C) and subsequently conducted hierarchical clustering on the dissimilarity matrix to aggregate strongly associated genes into the same module (Figure S9A). We found 15 modules across the 8 stages of wheat spikelet development (Figure 4A and Figure S9A). The MEred module and the MEblack module exhibited the strongest relationships with the DR stage and the FM stage, respectively (Figure 4A).

Differential gene analysis of bulk RNA‐seq and WGCNA data indicated that the DEGs in C4 of k‐means clustering demonstrated significantly elevated expression levels during the DR stage relative to other phases. The DEGs in C5 were exclusively strongly expressed during the FM stage. The results indicate that the DEGs in C4 and C5 may signify stage‐specific genes for the DR and FM stages, respectively (Figure 3C). The establishment of a weighted gene co‐expression network during wheat spike formation revealed that the MEred module is most closely linked to the DR stage, whereas the MEblack module is most closely linked to the FM stage. This suggests that the MEred and MEblack modules may function as stage‐specific components for the DR and FM stages, respectively (Figure 4A). The results indicate that the genes in C4 and the MEred module may contribute to the morphological development of the spikelet primordium in wheat spikes, whereas the genes in C5 and the MEblack module may be implicated in the differentiation of floret primordium morphology in wheat spikes.

To ascertain pivotal genes implicated in the morphological development of spikelet and floret primordia in wheat, we examined the intersections between 89 DEGs in the C4 group and 4383 expressed genes in the MEred module, alongside the intersections between 95 DEGs in the C5 group and 2944 expressed genes in the MEblack module. Our research indicated that eight genes were co‐expressed in both the C4 group and the MEred module, whereas 117 genes were co‐expressed in both the C5 group and the MEblack module (Figure S9B,C). Employing the established weighted gene co‐expression network created during the wheat spike development phase, we computed the weights of these 8 genes and 117 genes within the MEred and MEblack modules, respectively. The associated gene regulatory networks were depicted with Cytoscape software. The findings revealed that four genes, *TraesCS2A02G127400*, *TraesCS4D02G262000*, *TraesCS6B02G408500*, and *TraesCS6B02G408400*, serve as hub genes during the DR phase of wheat spikes. Furthermore, five genes, including *TraesCS5B02G277200*, *TraesCS7B02G257400*, *TraesCS2A02G532700*, *TraesCS3D02G427500*, and *TraesCS2A02G394500*, are recognized as hub genes during the FM stage of wheat spikes (Figure 4B,C).

**FIGURE 5:**
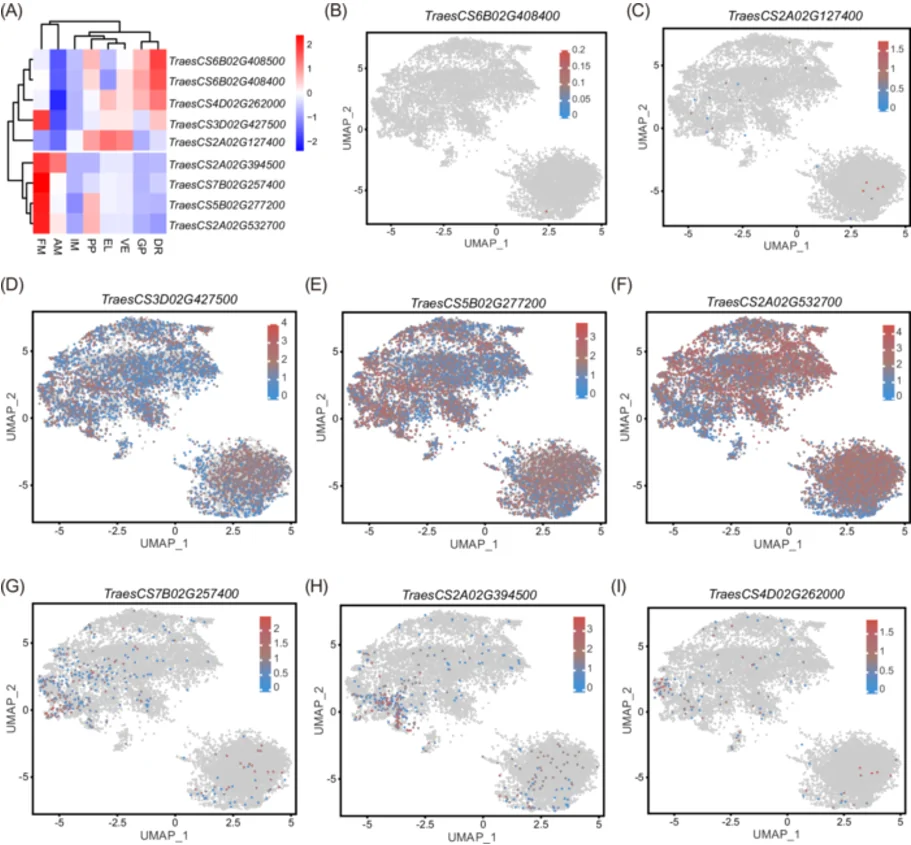
Expression of hub genes throughout wheat spike development and in cell types. (A) Expression variations of nine hub genes across eight developmental stages of wheat spike. AM, anther meristem stage; DR, double ridge; EL, elongation stage; FM, floral meristem stage; GP, glume primordium differentiation stage; IM, inflorescence meristem stage; PP, pistil primordium stage; VE, vegetative stage. (B−I) Distribution of eight hub genes in UMAP mapping. The hue of each dot signifies the expression level of the respective gene within the cell. The color gray signifies that the gene is not expressed in this cell.

The expression variations of the nine hub genes during the eight developmental stages of wheat spikes revealed that the expression levels of *TraesCS4D02G262000*, *TraesCS6B02G408500*, and *TraesCS6B02G408400* reached their zenith at the DR stage. The peak expression levels of *TraesCS5B02G277200*, *TraesCS7B02G257400*, *TraesCS2A02G532700*, *TraesCS3D02G427500*, and *TraesCS2A02G394500* were recorded during the FM stage. Furthermore, *TraesCS2A02G127400* demonstrated its peak expression level at the EL stage (Figure 5A). Additionally, the expression profiles of these nine genes in the cell types of wheat spike scRNA‐seq were examined, employing UMAP to illustrate their expression properties. The results indicated that, except for *TraesCS6B02G408500*, which was undetected in the scRNA‐seq cell types, the other eight genes were found across many cell types. Significantly, *TraesCS6B02G408400* and *TraesCS2A02G127400* exhibited expression solely in a restricted subset of cells (Figure 5B,C), whereas *TraesCS3D02G427500*, *TraesCS5B02G277200*, and *TraesCS2A02G532700* demonstrated expression throughout all tissues of the wheat spike (Figure 5D−F). *TraesCS7B02G257400*, *TraesCS2A02G394500*, and *TraesCS4D02G262000* exhibited prominent expression in the meristem, promeristem, protoxylem, and protophloem (Figure 5G−I). Of the nine hub genes, *TraesCS7B02G257400*, *TraesCS2A02G394500*, and *TraesCS4D02G262000* demonstrated tissue‐specific expression in wheat spikes (Figure 5B−I).

### Subgenomic asymmetrical expression during wheat spike development

**FIGURE 6:**
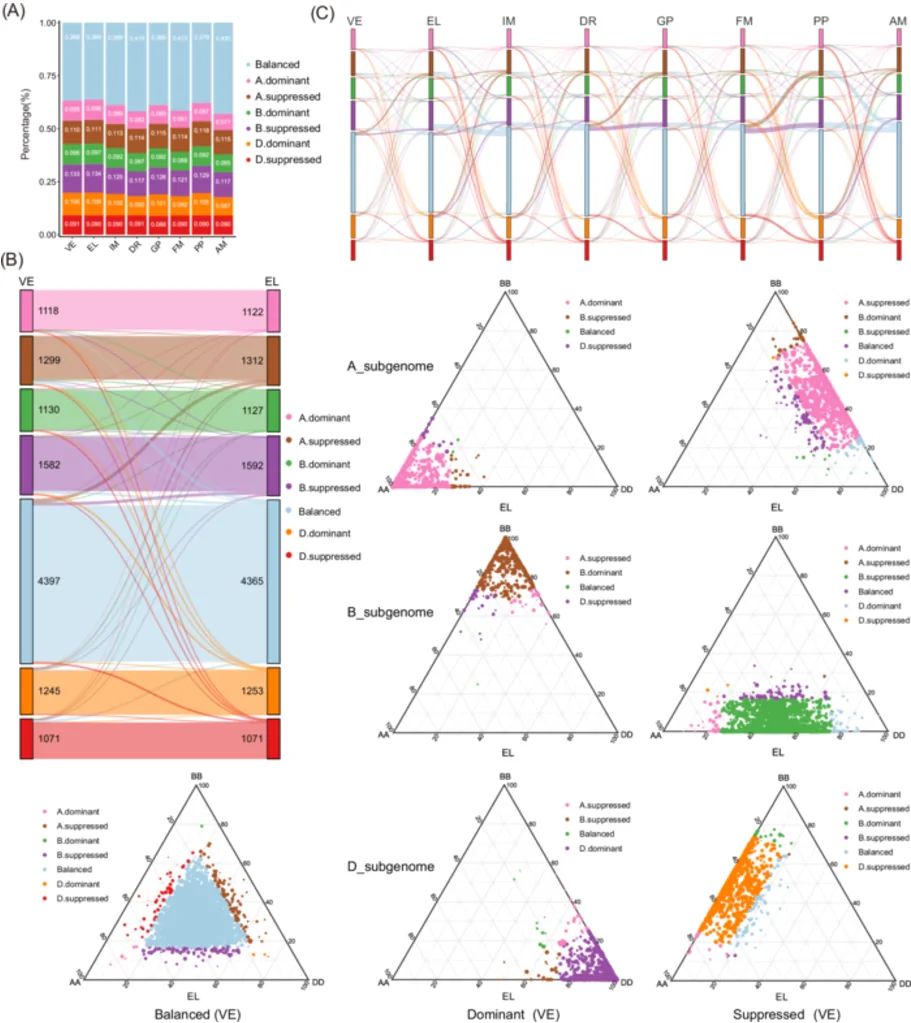
Homeolog in different periods asymmetrical expression in syntenic homoeolog triads. (A) Histograms represent the proportion of homologous gene expression deviation types at each developmental period; the vertical axis represents the proportion of each type. (B) Changes in the asymmetrical expression patterns from VE to EL. Sankey diagram represents the changes of homologous gene asymmetrical expression types in two periods. Ternary diagram represents the changes of seven homologous gene asymmetrical expression types. The dispersal of genes in the ternary diagram signifies the nature of homologous gene expression during the EL period, whereas the diagram is marked with the corresponding kind from the VE phase; A, B, and D represent the three genomes of wheat. (C) Changes in the type of homologous gene asymmetrical expression during various periods of wheat spike development. EL, elongation stage; VE, vegetative stage.

In this study, we investigated the transcriptomes across eight stages of wheat spike development to assess the preferential expression of homologous genes (Figure 6A−C and Figures S10−S16). Our findings revealed that the majority of triads were classified as balanced throughout each developmental stage. The proportion of balanced triads ranged from 43% during the AM to 36.4% in the EL (Figure 6A). Notably, the prevalence of suppressed‐type triads was significantly higher than that of dominant‐type triads across all eight developmental phases. The D. suppressed triads exhibited minimal variation, maintaining approximately 9% throughout the formation of the wheat spike. In contrast, during the development of immature wheat spikes, both dominant and suppressed triads from subgenomes A and B demonstrated greater variability. The highest proportions of balanced triads were observed at the DR, FM, and AM, with percentages of 41.9%, 41.5%, and 43%, respectively (Figure 6A and Figure S10).

The aforementioned approaches provide a fixed perspective on the comparative asymmetrical expression of homologous genes within a single sample. Conversely, the growth of the wheat spike is a dynamic and evolving process. To gain a deeper understanding of how the expression of similar genes varies throughout this process, we investigated the different types of asymmetrical expression among the same groups of genes at various stages of wheat spike development (Figure 6B and Figures S11−S16). Our comparative analysis of consecutive developmental periods revealed that approximately 80% of the triads exhibited consistency throughout development. Only a small number of triads displayed changes in their asymmetrical expression as the spike progressed. Among the triads that underwent alterations in asymmetrical expression, balanced triads demonstrated the capacity to transition into any of the seven types of homologous gene asymmetrical expression. Suppressed triads frequently shifted to the adjacent category but did not transition to the opposite category. In contrast, dominant triads exclusively transformed into the neighboring category (Figure 6B and Figures S11−S16).

Subsequently, we analyzed the fluctuating patterns of gene expression variations in homologous genes across different stages of wheat spike development (Figure 6C). The results revealed that dominant‐type triads were highly conserved throughout the growth of the wheat spike, while frequent transitions occurred between suppressed and balanced‐type gene triads. A significant proportion of suppressed gene triads transitioned to a balanced state during various developmental stages, specifically from the EL to the IM, from the IM to the DR, from the GP to the FM, and from the PP to AM. Conversely, a considerable number of balanced gene triads transformed into suppressed‐type triads during the transitions from the DR to the GP, and from the GP and FM to the PP. Furthermore, the changes in each type of triad from the VE to the EL were more evenly distributed (Figure 6C).

### Single‐cell resolution of subgenomic asymmetrical expression in wheat spike

**FIGURE 7:**
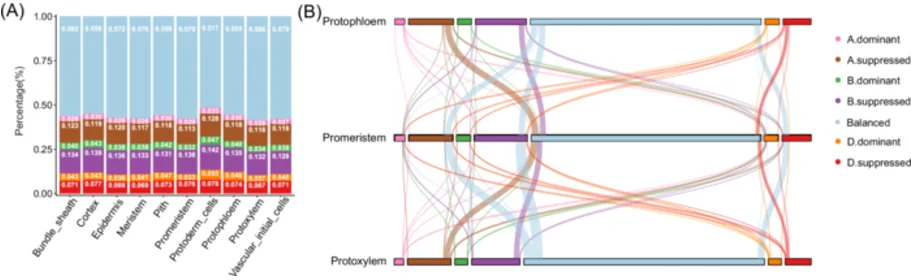
Subgenomic asymmetrical expression of various cell types in wheat spikes. (A) Histograms represent the proportion of homologous gene expression deviation types at each cell type in wheat spikes; the vertical axis represents the proportion of each type. (B) Changes in the type of homologous gene asymmetrical expression from promeristem to protophloem and protoxylem.

To assess the differences in homologous gene expression among various cell types in wheat spikes, we analyzed gene expression deviations across 10 distinct cell types (Figure S17). Our findings indicate that the percentage of dominant gene triplets is consistently low, ranging from 2.6% to 4.7% across all cell types. In contrast, suppressed gene triplets are more prevalent, with frequencies ranging from 6.7% to 15%. Balanced gene triplets exhibit the highest proportion, varying from 48.3% to 57.9%. Notably, no significant differences were observed in the frequency of dominant gene triplets among the subgenomes across different cell types. However, the frequency of D. suppressed gene triplets exhibited slight variation and was significantly lower than that of A. suppressed and B. suppressed gene triplets. Conversely, the frequencies of A. suppressed and B. suppressed gene triplets varied considerably among the different cell types (Figure 7A and Figure S17).

In contrast to the dominant gene triplet, which primarily transitioned to neighboring categories during the differentiation process from the promeristem to the protophloem and protoxylem, the suppressed gene triplet exhibited a significantly greater diversity of transition types. Furthermore, while variations in gene expression were observed during spike development, the balanced gene triplet predominantly shifted towards the suppressed gene triplet. This phenomenon can be attributed to the relatively smaller number of differential genes involved in cell differentiation, which allows for a clearer representation of significant differences (Figures S18 and Figure S19). Notably, during cell development, the majority of variations in gene triad expression were observed between the balanced and suppressed types (Figure 7B).

## 3 DISCUSSION

### Cell types of wheat spikes

The scRNA‐seq has been extensively applied in medical and animal studies; however, research on single‐cell analysis in plants remains in its nascent stages. This is primarily due to the presence of cell walls in the outer layer of plant cells, which complicates the separation and capture of individual cells. Currently, studies on unicellular plants predominantly focus on species such as Arabidopsis, rice, maize, tomato, peanut, tobacco, poplar, and algae [20]. Notably, there is a lack of research on the transcriptome sequencing of wheat spikes unicells. Previous investigations have shown that the shoot apical meristem, located at the apex of the stem, produces three main meristematic tissues in the juvenile spike tissues of wheat: the procambium, the ground meristem, and the protoderm. The main formation layer is responsible for generating protoxylem, fascicular cambium, and protophloem, which are essential types of vascular tissue [61, 62]. The ground meristem contributes to the formation of the pith and cortex, with the pith giving rise to the pith rays, which subsequently develop into the interfascicular cambium. Meanwhile, the protoderm forms the outermost layer of cells known as the epidermis. Cellular annotation represents a critical and challenging step in the analysis of the transcriptome of individual plant cells, particularly in non‐model plant applications, as it lays the groundwork for subsequent data processing [63, 64, 65, 66].

In this study, we performed scRNA‐seq on common wheat spikes at the DR. We identified and categorized 5989 high‐quality cells into 13 distinct groups (Figure 1A and Figure S3). To accurately determine the cell types within these clusters, we analyzed the expression of genes known to serve as markers for specific cell types across all 13 clusters. Initially, we confirmed the presence of meristem, promeristem, and protoxylem by examining the expression patterns of these marker genes. Subsequently, we conducted functional analyses on the remaining clusters, focusing on genes that exhibited high expression levels. We also evaluated the expression of corresponding genes in various tissues of Arabidopsis, rice, and maize, which further validated the presence of epidermis and bundle sheath. Finally, utilizing the information derived from cellular descending clustering and the tissue types of wheat spikes, we classified the 13 clusters into 10 distinct cell types (Figure 1C). The subsequent RNA in situ hybridization experiments and data mining provided direct or indirect evidence supporting the validity of the cell type classifications made in this study (Figure S4). This suggests that the first single‐cell transcriptome map of wheat spikes, as presented here, offers significant reference value for the functional analysis of wheat spikes.

The number of scRNA‐seq studies in the field of plant sciences has markedly increased in recent years, underscoring the growing recognition of single‐cell sequencing as a method capable of elucidating cellular states across multiple molecular levels. Furthermore, the rapidly expanding domain of single‐cell biology is witnessing broader applications. For instance, the integration of single‐nucleus RNA sequencing with single‐nucleus assays for chromatin accessibility sequencing (snATAC‐seq) facilitates the establishment of correlations between chromatin accessibility and gene expression at the single‐cell level in specific cell types within Arabidopsis roots [24]. The advancement of single‐cell multi‐omics technologies, such as the Chromium Single‐Cell Multiome ATAC + Gene Expression platform developed by 10× Genomics in Pleasanton, USA, enables the simultaneous investigation of the genome, epigenome, transcriptome, proteome, and/or metabolome [20, 67, 68, 69, 70]. This integrated multi‐omics analysis yields a more comprehensive understanding of the transcriptional control of cellular states, interactions, and behaviors compared to single‐cell transcriptomics alone. Moreover, the development of scalable, multimodal single‐cell analysis methodologies that incorporate histology and imaging technologies will enhance our understanding of how genotype influences phenotype at the individual cell level [71, 72].

### Asymmetrical subgenomic expression during wheat spike formation

Polyploids arise through either whole genome duplication or interspecific hybridization and are prevalent across various eukaryotic plant and fungal lineages. The phenotypic diversity observed in many of the world's crucial crops is attributed to the coordinated expression of closely related homologous genes within polyploid organisms. These interactions yield a range of outcomes, including buffering effects that occur when gene homologs exhibit overlapping functions, and dominance effects that emerge when mutations in a single homolog led to a dominant phenotype [73, 74]. Understanding the implications of these interactions on gene expression will provide essential insights for enhancing crops through the targeted manipulation of one or more homologous genes, thereby enabling precise control of phenotypic responses [1]. *T. aestivum* has undergone two episodes of polyploidization, resulting in a genome that is both extensive and complex.

To investigate the patterns of gene expression during the development of the wheat spike, we analyzed the preferential expression of transcripts across eight distinct developmental stages and homologous genes in ten different cell types within the spike (Figures 6, 7, and Figures S10−S19). Our findings revealed a lower incidence of gene triads exhibiting asymmetrical expression patterns at the individual cell level compared to those identified through bulk RNA sequencing (Figures 6A and 7A). Notably, promeristem tissues demonstrated heightened levels of asymmetrical expression patterns, suggesting that imbalanced gene expression may play a role in cell differentiation (Figure 7A). Before the wheat spike development reaching the DR, FM, and AM, a significant number of repressive gene triads transitioned into balanced gene triads. Subsequently, these balanced gene triads reverted to repressive gene triads. Throughout the development of the spike, the dominant gene triads remained relatively stable, indicating that the reciprocal transformation between repressive and balanced gene triads is a critical factor influencing spike formation (Figure 6C). These findings underscore the importance of the transition from repressive to balanced gene triads in spike development.

Gene expression profiles from 850 wheat tissues and developmental contexts reveal that over 75% of the genes are active in at least one tissue, with approximately 30% of the gene triads displaying asymmetrical expression across the three subgenomes [75]. Recent studies have shown that a significant proportion of evenly matched genes identified through bulk RNA‐seq in wheat roots exhibit preferential expression at the individual cell level [51]. Furthermore, inter‐subgenomic gene translational asymmetry is commonly observed throughout grain development in wheat [76]. One likely mechanism contributing to this asymmetry is the variation in mRNA sequence characteristics and translational regulatory elements. Despite the potential of polyploidy to profoundly influence gene expression, our comprehension of the similarities and differences in expression patterns among homologous genes, as well as the spatial and temporal dynamics of these relationships and the effects of interactions between individual homologous genes on biological traits, remains limited [8].

## 4 CONCLUSION

The present study utilized DR_JM spikes of common wheat JM22 to generate high‐fidelity scRNA‐seq data. As a result, a comprehensive single‐cell transcriptome atlas of the wheat spike was constructed. Furthermore, the analysis of homologous gene expression biases across transcriptomes and cell types during wheat spike development was conducted by integrating bulk RNA‐seq data from eight distinct stages. This analysis suggests that asymmetrical gene expression may act as a catalyst for cell differentiation, and the transition of gene triplets from suppressed to balanced forms is a crucial determinant of spike differentiation.

## 5 METHODS

### Plant material

In this investigation, the main cultivar in the Huanghuai wheat region of China, Jimai 22 (JM22), which is noted for its strong stress resistance and broad adaptability, was used as the experimental material. The wheat seeds underwent surface sterilization using a 70% ethanol solution, followed by three rinses with sterile water, before being placed on filter paper to initiate germination. To accelerate the formation of DR and FM, ensuring the synchronized development of experimental materials. Vernalization was conducted in a controlled indoor greenhouse setting, following the procedure outlined by Lewis et al. (2008) [77]. Once the green shoots had sprouted, the seeds were relocated to a refrigerator maintained at 4°C for a duration of 6 weeks to break the dormancy of winter wheat by inducing the expression of flowering‐related genes through low temperatures, thereby promoting the transition of IMs to reproductive growth. The vernalized seedlings were then transferred to a growth room with a constant temperature of 24°C, a light period of 16 h, and a humidity level of 40%. RNA was isolated from samples collected at six distinct developmental stages: EL, IM, DR, GP, FM, and PP, using a stereomicroscope (Leica S6E) (Figure S20).

### Protoplast isolation for wheat spike

A Leica S6E stereomicroscope was utilized to obtain a sample of common wheat JM22 spike at the DR. The sample was immediately immersed in an enzymatic solution consisting of 0.1% bovine serum albumin, 10 mM CaCl_2_, 10 mM KCl, 0.1 M 4‐morpholineethanesulfonic acid, 1.5% macerozyme R10, 0.4 M mannitol, and 1.5% pectolyase Y23, adjusted to a pH of 5.7, and subsequently subjected to centrifugation. During the enzymatic hydrolysis of the wheat spike, a vacuum treatment was applied for 30 min, followed by incubation at 28°C. In the initial phase of enzymatic digestion, the rotational speed was maintained between 60 and 70 rpm for 2 h. This was followed by an increase in rotational speed to 90 rpm for an additional 0.5 h of enzymatic digestion. After the digestion process, the reaction mixture was removed, and an equivalent volume of W5 buffer (154 mM NaCl, 5 mM KCl, 10 mM CaCl_2_, and 2 mM MES; pH 5.7) was added to halt the reaction. The mixture was then filtered through a 300‐mesh screen, and the resulting liquid was carefully poured into a petri dish at an angle before being transferred to a 10 mL tube. The initial sample was subjected to centrifugation at a force equivalent to 130 times the acceleration due to gravity, with both acceleration and deceleration rates set at 1, for a duration of 5 min. Following this process, the liquid portion above the sediment was carefully removed. Protoplasts were then resuspended in an 8% mannitol solution and subjected to a second centrifugation under the same conditions to eliminate the upper layer of liquid. A suitable volume of the protoplast solution was obtained and gently mixed with an equal volume of 8% mannitol. Subsequently, a specific volume of this mixture was extracted and combined with a 0.4% Trypan Blue solution in a 9:1 ratio. The Countess® II Automated Cell Counter was employed to determine the total cell count and the percentage of viable cells. It is essential to ensure that the proportion of viable cells is at least 90% when increasing the cell concentration to 1000 cells/μL or higher.

### scRNA‐seq library construction and sequencing

A volume of 100 μL of a suspension containing individual cells was combined with gel beads that contained barcode information to produce single‐cell gel beads in emulsion, referred to as Gel Bead‐In‐Emulsions (GEMs). Following this, the gel beads were disrupted to release the captured sequences, which included the barcode sequences. Subsequently, the cDNA fragments underwent reverse transcription. The gel bead fragmentation process entails the disintegration of the oil droplets, after which cDNA serves as a template for PCR amplification. The library was constructed using the Chromium Controller and the Chromium Single Cell 3' Reagent Kit (V.3.0.0, 10× Genomics). The scRNA‐seq library was generated with the Chromium single‐cell 3' gel bead and library kit (P/N: 120236, 120237, 120262). This library underwent paired‐end sequencing on an Illumina Hiseq. 4000 sequencer. The raw scRNA‐seq data set comprises three components: Read1, Read2, and the i7 index read. Read1 has a length of 26 base pairs (bp) and includes a sequence consisting of a 16 bp 10× Barcode and a 10 bp UMI. These sequences are located in the 3' UTR region and are utilized for gene identification. Read2 has a length of 98 base pairs and contains cDNA sequences. All products from the GEMs are pooled together to create a sequencing library suitable for high‐throughput sequencing, ultimately resulting in the generation of a library for single‐cell gene expression data.

### Data quality control and gene expression quantification of scRNA‐seq

Cell Ranger is a suite of analysis pipelines developed by 10× Genomics specifically for processing scRNA‐seq data generated using their Chromium platforms. The genomic sequences of *T. aestivum* were obtained from publicly accessible databases (http://plants.ensembl.org/Triticum_aestivum/Info/Index) and utilized as the reference genome [37]. The raw scRNA‐seq data set was analyzed using Cell Ranger (v.6.0.1, 10× Genomics) with default parameters [78]. This analysis involved several steps: cellranger mkfastq for demultiplexing raw base call files, cellranger count for alignment and generation of the gene‐cell matrix, cellranger aggr for aggregating data from multiple samples, and cellranger reanalyze for additional analyses. Only uniquely mapped reads with a MAPQ value of 255 were used to generate a UMI count matrix, wherein each row corresponds to a single gene and each column represents a cell. The UMI counting and cell barcode calling processes resulted in the generation of cell‐by‐gene matrices. Each sample's cell‐by‐gene matrix was subsequently imported into the Seurat (v.3.1.1) program for further analysis [79]. The chloroplast (NC_002762) and mitochondrial (NC_036024) genomes were sourced from the NCBI database and analyzed individually to assess sequencing quality [80, 81]. Protoplasts with mitochondrial gene contributions exceeding 1% and chloroplast gene contributions exceeding 5% were filtered out.

### Cell clustering, annotation, and marker gene selection

Cells with UMI counts exceeding 50,000 or falling below 500 were excluded from further analysis using the EmptyDrops technique [82]. Additionally, potential doublet cells were removed with the aid of DoubletFinder (v.2.0.2), which also excluded cells exhibiting gene expression counts below 200 or above 10,000 [83]. The “LogNormalize” technique was employed to normalize gene expression on a per‐cell basis. To address the influence of batch effects and other behavioral factors on clustering, the “Harmony” technique was utilized [84]. The Louvain method was subsequently applied for cell clustering [85]. The “FindNeighbors” function was used to construct the Shared Nearest Neighbor graph, with dimensions ranging from 1 to 49. Following this, the “FindClusters” function was executed to cluster the cells at a resolution of 0.8. Principal component analysis was utilized to address the complexity and high dimensionality of the data. Subsequently, the aforementioned clustering results were transformed into two‐dimensional and three‐dimensional representations using two nonlinear dimensionality reduction techniques: UMAP and *t*‐Distributed Stochastic Neighbor Embedding. Finally, the “RunTSNE” and “RunUMAP” functions were performed to visually represent the results using nonlinear dimensional reduction techniques.

### Pseudo‐time analysis

To investigate the process of cell differentiation and the molecular mechanisms governing cellular fate, we employed Monocle (v.2.20.0) for pseudo‐time analysis [86]. Initially, we identified genes with *p*‐adjusted values below 0.05 as marker genes specifically associated with developmental processes. We then set the max_components parameter to 2 and used “DDRTree” for dimensionality reduction, resulting in a reduction to two components. The “orderCells” function was applied in the low‐dimensional space to arrange the cells in a pseudo‐temporal order based on transcriptome correlations. Additionally, we utilized the “plot_cell_trajectory” function to visualize cell trajectories. To establish the starting point in the trajectory, we executed the “orderCells” function with the root_state specified. The “BEAM” function was employed to analyze genes dependent on specific branches, and subsequently, the “plot_genes_branched_heatmap” function was used to visualize the differences in gene expression between the two branches.

### Sequence alignment and differential gene expression analysis of bulk RNA‐seq

We independently compiled and gathered transcriptome sequencing data for eight developmental stages of wheat spike: VE, EL, IM, DR, GP, FM, PP, and AM. The bulk RNA‐seq raw sequencing data were sourced from major genomic databases, including National Center for Biotechnology Information (NCBI), European Molecular Biology Laboratory, and China National Center for Bioinformation, utilizing the accession numbers provided in the relevant transcriptome literature (Table S4) [87, 88, 89]. The reference genomic data for wheat were obtained from the Ensembl database (https://plants.ensembl.org/Triticum_aestivum/Info/Index). Given that the transcriptome data originated from various species and experimental platforms, we conducted quality control on the raw data to filter, compare, and quantify it. The NGS QC Toolkit (v.2.3.3) was employed to analyze the raw sequencing data [90]. Subsequently, we utilized the Hisat2 program to align the cleaned reads to the wheat reference genome [91]. To address batch effects, we applied the “removeBatchEffects” function from the limma software package, with all parameters set to their default values. The mRNA FPKM values were calculated using edgeR and StringTie software, while expression disparities across samples were assessed using the DESeq. 2 utility in R [92, 93]. Additionally, we employed the k‐means clustering technique to partition the time‐series transcriptome data into distinct clusters.

### GO enrichment analysis

The genes that exhibited differential expression, as identified through analysis with the edgeR package, along with the genes uniquely expressed in each cell cluster—determined using the “FindAllMarkers” function in the Seurat package—were submitted to AgriGO (v.2.0) for GO enrichment analysis [94]. Test statistics for each cluster were adjusted by multiplying by the sign of the log_2_fold‐change value relative to the reference panel. GO terms with gene sets smaller than 10 or larger than 600 were excluded from the analysis. Terms were deemed significantly enriched at a false discovery rate of less than 0.05, following 10,000 permutations. Subsequently, GO enrichment analysis was performed on the genes within each cluster, employing ClusterGV (https://github.com/junjunlab/ClusterGVis) is software for visualization.

### Subgenome asymmetrical expression analysis

We compiled a total of 20,400 homologous gene triads from the Triticeae‐GeneTribe database (http://wheat.cau.edu.cn/TGT/m16/?navbar=Homologues), encompassing 61,200 genes. To maintain consistency in the relative expression levels of each homologous gene within the triads, we normalized the absolute Transcripts Per Kilobase of exon model per million mapped reads values for each gene through a procedure that ensured their sum equaled 1.0 in each sample.

We calculated the Euclidean distance for each triad by normalizing the data and subsequently categorized these triads into several groups based on homologous gene asymmetrical expression, determined by the shortest distance. The categories include balance, A. dominant, A. suppressed, B. dominant, B. suppressed, D. dominant, and D. suppressed. This classification is consistent with the categories established in previous research [8, 51]. Additionally, we visually represented the asymmetrical expression of the triplex subgenome using the ggtern (v.3.3.5) package in the R programming language [95].

### Real‐time quantitative PCR (qPCR) analysis

Total RNA was extracted from wheat spikes using the TRIzol reagent (Invitrogen, No. 15596026). Subsequently, complementary DNA (cDNA) was synthesized employing the One‐Step gDNA Removal and cDNA Synthesis SuperMix (TransGen Biotech, No. AE311–02). qPCR was performed utilizing the CFX Connect system from Bio‐Rad, in conjunction with the 2 × Universal SYBR Green Fast qPCR Mix (ABclonal, No. RK21203). The primers employed for qPCR are detailed in Table S5.

### RNA in situ hybridization

The T7 promoter sequence was incorporated into both the forward and reverse primers (Table S3) to amplify the coding region of a gene and generate a template. This template was then mixed with T7 RNA polymerase (Roche, No. 10881767001) to produce a digoxigenin‐labeled RNA probe. RNA in situ hybridization was conducted following the methodology previously described by Yang et al. (2021) [96]. Wheat spikes were collected and preserved in formaldehyde. Paraffin‐embedded samples, sectioned to a thickness of 10−12 μm, were placed on pre‐coated glass slides. The slides containing the sample sections underwent treatment with Histoclear to eliminate the wax, followed by digestion with Proteinase K (Roche, No. 03115828001). A series of ethanol gradients, ranging from lower to higher concentrations, including 100% ethanol, was then applied to remove moisture from the slides. The prepared slides with sample slices were hybridized with the digoxigenin‐labeled RNA probe. After incubation with Anti‐Digoxigenin‐AP Fab fragments (Roche, No. 11093274910), the slides were rinsed, and the signal was detected using an NBT/BCIP Stock Solution (Roche, No. 11681451001).

## AUTHOR CONTRIBUTIONS

**Fang He**: Funding acquisition; writing—original draft; software; writing—review and editing; methodology; visualization; data curation. **Xiaojuan Liu**: Writing—original draft; validation; investigation; writing—review and editing. **Qian Ma**: Validation; writing—review and editing. **Wei Wan**: Formal analysis; project administration; validation; writing—review and editing. **Luhua Li**: Supervision; project administration; resources; data curation; writing—review and editing. **Kuiyin Li**: Supervision; writing—review and editing. **Zhenzhen Jia**: Data curation; writing—review and editing. **Suqin Zhang**: Writing—review and editing. **Ruhong Xu**: Project administration; resources; writing—review and editing. **Mingjian Ren**: Funding acquisition; conceptualization; methodology; data curation; writing—review and editing.

## CONFLICT OF INTEREST STATEMENT

The authors declare no conflicts of interest.

## ETHICS STATEMENT

No animals or humans were involved in this study.

## Supporting information


**Figure S1:** DR_JM scRNA‐seq sequencing report.
**Figure S2:** Highly variable genes for subsequent analysis.
**Figure S3:** Single‐cell transcriptome clustering analysis of wheat spike.
**Figure S4:** RNA in situ hybridization of Marker genes in young wheat spike.
**Figure S5:** Trajectory analysis of GO enrichment of DEGs in each Cluster.
**Figure S6:** Differentially expressed gene data statistics.
**Figure S7:** qRT‐PCR validation of transcriptional changes of 54 genes screened from DEGs.
**Figure S8:** Gene co‐expression network.
**Figure S9:** Hub gene screening for wheat spike development.
**Figure S10:** Homeolog in different periods asymmetrical expression in syntenic homeologs triads.
**Figure S11:** Changes in the asymmetrical expression patterns from EL to IM.
**Figure S12:** Changes in the asymmetrical expression patterns from IM to DR.
**Figure S13:** Changes in the asymmetrical expression patterns from DR to GP.
**Figure S14:** Changes in the asymmetrical expression patterns from GP to FM.
**Figure S15:** Changes in the asymmetrical expression patterns from FM to PP.
**Figure S16:** Changes in the asymmetrical expression patterns from PP to AM.
**Figure S17:** Homeolog in different cell types asymmetrical expression in syntenic homeologs triads.
**Figure S18:** Changes in the asymmetrical expression patterns from promeristem to protophloem.
**Figure S19:** Changes in the asymmetrical expression patterns from promeristem to protoxylem.
**Figure S20:** Wheat spike at various developmental periods.

**Table S1:** Single cell data set quality.
**Table S2:** Results of cell subpopulation classification.
**Table S3:** Primer sequences for RNA in situ hybridization.
**Table S4:** Transcriptome data for each period of wheat spike development.
**Table S5:** qPCR primer sequences.

## Data Availability

The data that support the findings of this study are openly available in Genome Sequence Archive in BIG Data Center at https://bigd.big.ac.cn/, reference number PRJCA036262. The raw sequencing data of scRNA‐seq generated from this study have been deposited in the Genome Sequence Archive in BIG Data Center (https://bigd.big.ac.cn/), Beijing Institute of Genomics (BIG), Chinese Academy of Sciences, under the accession number: PRJCA036262 (https://ngdc.cncb.ac.cn/bioproject/browse/PRJCA036262). []The data and scripts used are saved in GitHub (https://github.com/hfwhere/DR_spike_2025). Supplementary materials (figures, tables, graphical abstract, slides, videos, Chinese translated version, and update materials) may be found in the online DOI or iMetaOmics Science http://www.imeta.science/imetaomics/.
