# Supplementary material for: The impact of asymmetrical gene expression on the development of spike morphology in Triticum aestivum
Source: IMetaOmics. 2025 Aug 27;2(3):e70047. doi: 10.1002/imo2.70047 (PMC12806133; doi:10.1002/imo2.70047)
Supplement: Supplementary file 1 — Figure S1: DR_JM scRNA‐seq sequencing report. Figure S2: Highly variable genes for subsequent analysis. Figure S3: Single‐cell transcriptome clustering analysis of wheat spike. Figure S4: RNA in situ hybridization of Marker genes in young wheat spike. Figure S5: Trajectory analysis of GO enrichment of DEGs in each Cluster. Figure S6: Differentially expressed gene data statistics. Figure S7: qRT‐PCR validation of transcriptional changes of 54 genes screened from DEGs. Figure S8: Gene co‐expression network. Figure S9: Hub gene screening for wheat spike development. Figure S10: Homeolog in different periods asymmetrical expression in syntenic homeologs triads. Figure S11: Changes in the asymmetrical expression patterns from EL to IM. Figure S12: Changes in the asymmetrical expression patterns from IM to DR. Figure S13: Changes in the asymmetrical expression patterns from DR to GP. Figure S14: Changes in the asymmetrical expression patterns from GP to FM. Figure S15: Changes in the asymmetrical expression patterns from FM to PP. Figure S16: Changes in the asymmetrical expression patterns from PP to AM. Figure S17: Homeolog in different cell types asymmetrical expression in syntenic homeologs triads. Figure S18: Changes in the asymmetrical expression patterns from promeristem to protophloem. Figure S19: Changes in the asymmetrical expression patterns from promeristem to protoxylem. Figure S20: Wheat spike at various developmental periods. [file IMO2-2-e70047-s001.docx]

**Supporting information to**

**The impact of** **asymmetrical gene expression on the development of spike morphology in *Triticum aestivum***

**Running title**: Asymmetrical gene expression influences spike development in *Triticum aestivum*

Fang He^1#^, Xiaojuan Liu^2#^, Qian Ma^1^, Wei Wan^1^, Luhua Li^1^, Kuiyin Li^2^, Zhenzhen Jia^1, 3^, Suqin Zhang^1^, Ruhong Xu^1^, Mingjian Ren^1^*

^1^Guizhou Subcenter of National Wheat Improvement Center, Key Laboratory of Functional Agriculture of Guizhou Provincial Higher Education Institutions, Guizhou University, Guiyang 550025, China

^2^Anshun University, Anshun 561000, China

^3^School of Life Sciences, Guizhou Normal University, Guiyang 550025, China

**^#^**These authors contributed equally: Fang He, Xiaojuan Liu.

*Correspondence: mxren@gzu.edu.cn (Mingjian Ren)


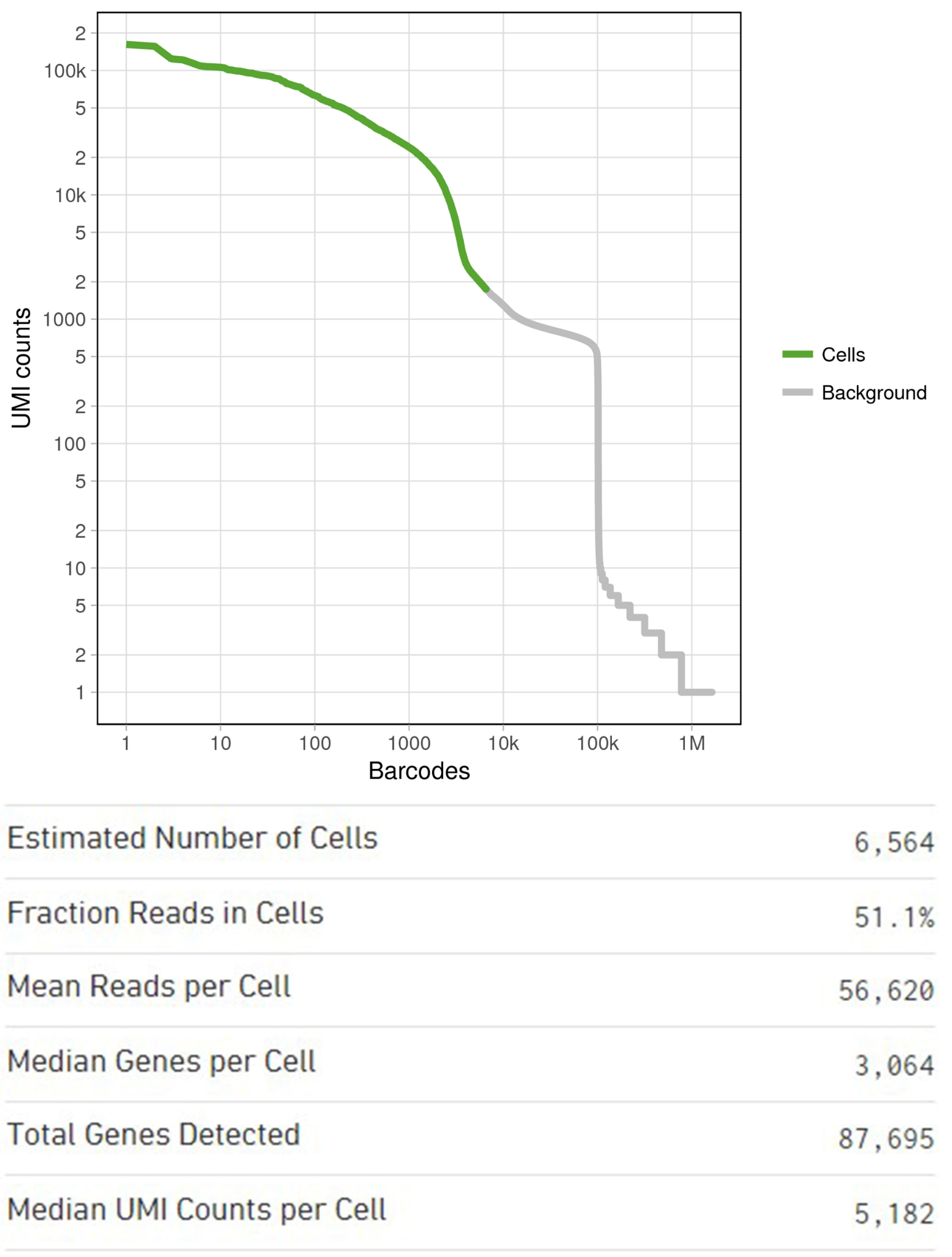


**Figure S1 DR_JM scRNA-seq sequencing report.**


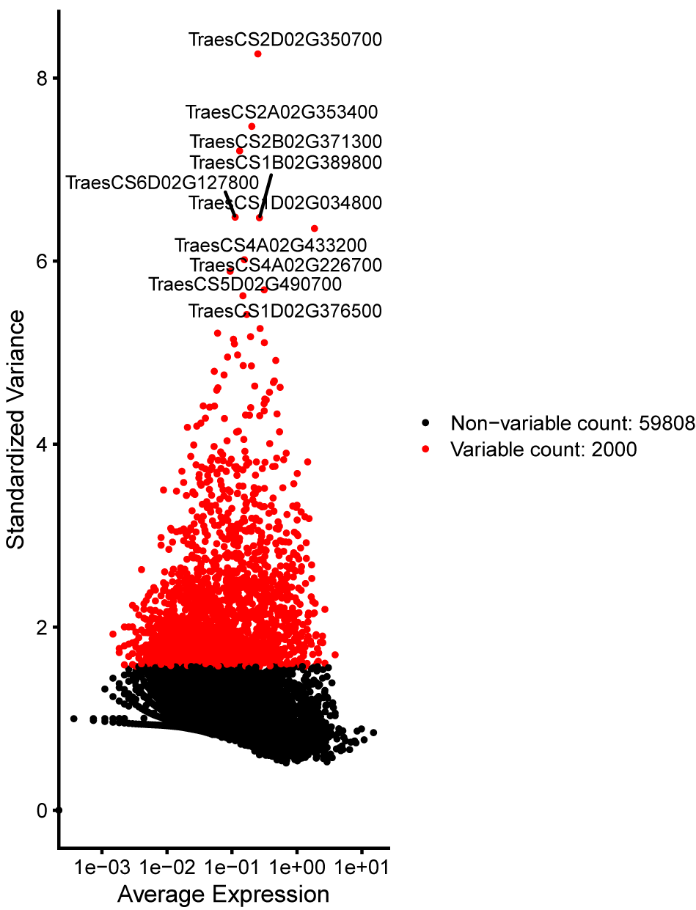


**Figure S2 Highly variable genes for subsequent analysis.**


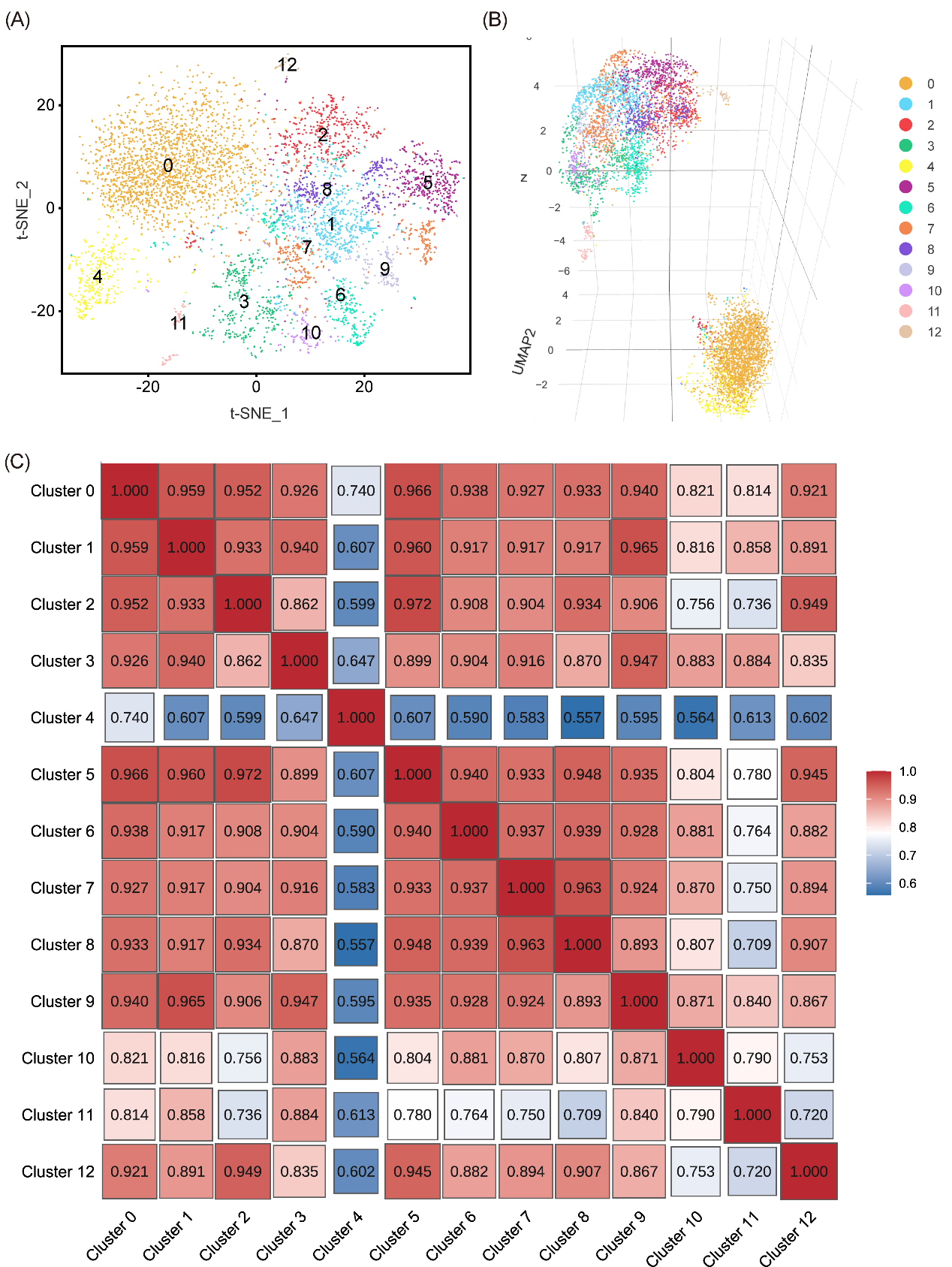


**Figure S3 single-cell transcriptome clustering analysis of wheat spike.** (A) single-cell cluster clustering *t*-SEN plot; (B) single-cell cluster clustering 3D-UMAP plot; (C) correlation between different clusters.


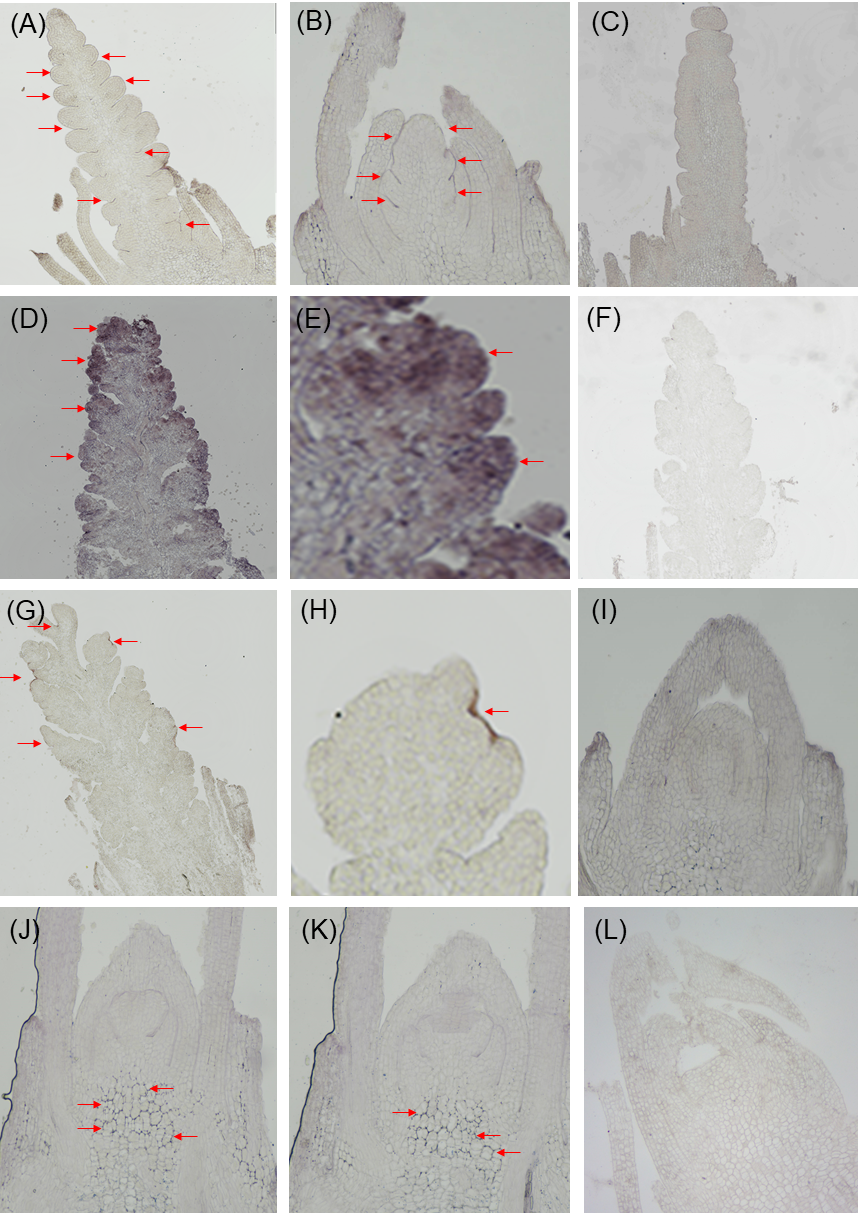


**Figure S4 RNA in situ hybridization of Marker genes in young wheat spike**. (A) Hybridization of *TraesCS6A02G063900* at the stage of glume primordium differentiation stage; (B) Hybridization of *TraesCS6A02G063900* at double ridge stage; (C) Justice probe of *TraesCS6A02G063900*; (D) Hybridization of *TraesCS4D02G205000* at the stage of floral meristem stage; (E) Local enlargement of the D picture; (F) *TraesCS4D02G205000*'s justice probe (G) *TraesCS2A02G404800*'s hybridization at the stage of floral meristem stage; (H) partial enlargement of G picture; (I) *TraesCS2A02G404800*'s justice probe; (J) *TraesCS4A02G392300*'s hybridization at single ridge stage; (K) *TraesCS4A02G392300* hybridization at single ridge stage; (L) *TraesCS4A02G392300* justice probe; the red arrows denote gene expression signals. Bars = 200 um


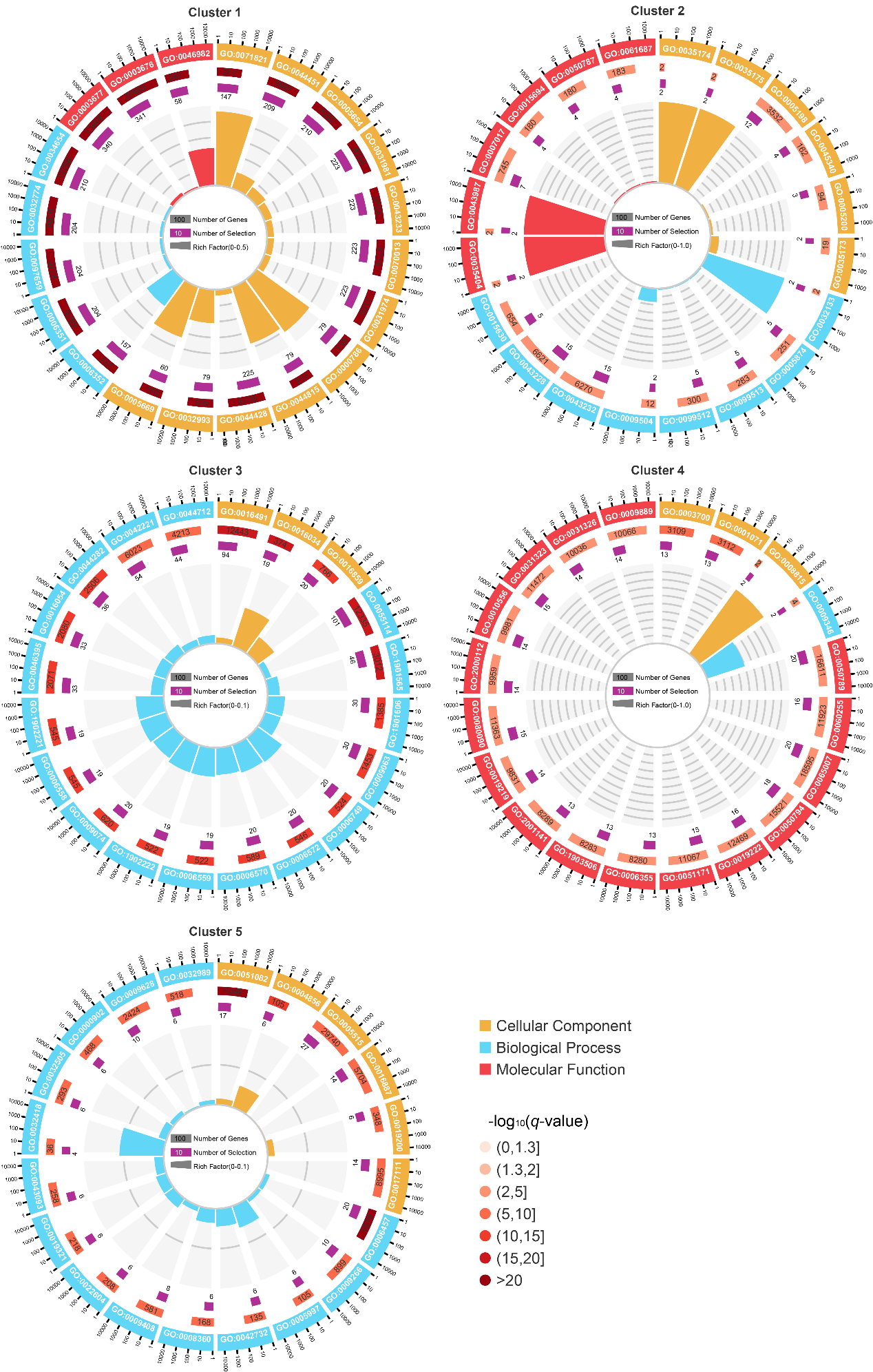


**Figure S5 trajectory analysis of GO enrichment of differentially expressed genes in each Cluster.** The outermost is a scale indicating the number of differential genes; the first circle indicates the ID of the GO term; the second circle indicates the number of genes enriched in the background gene set; the third circle indicates the number of genes enriched in the differential gene set; and the innermost is the Gene Ratio.


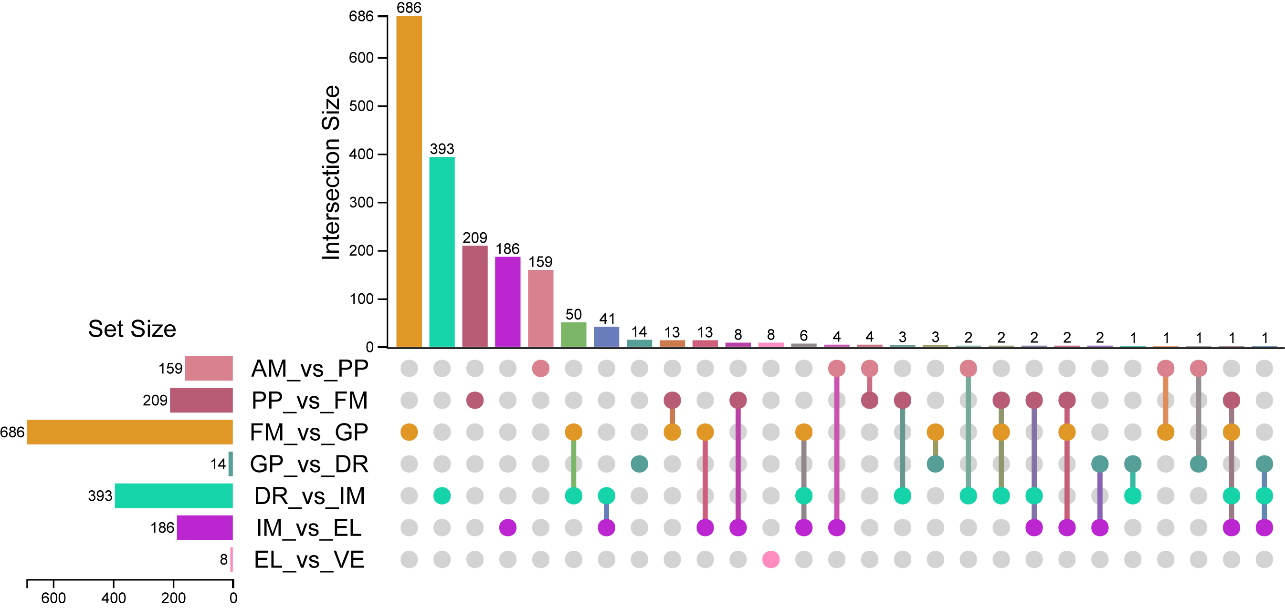


**Figure S6 Differentially expressed gene data statistics.** The horizontal axis represents the sample intersection area, and each column indicates the number of DEGs in the corresponding intersection.


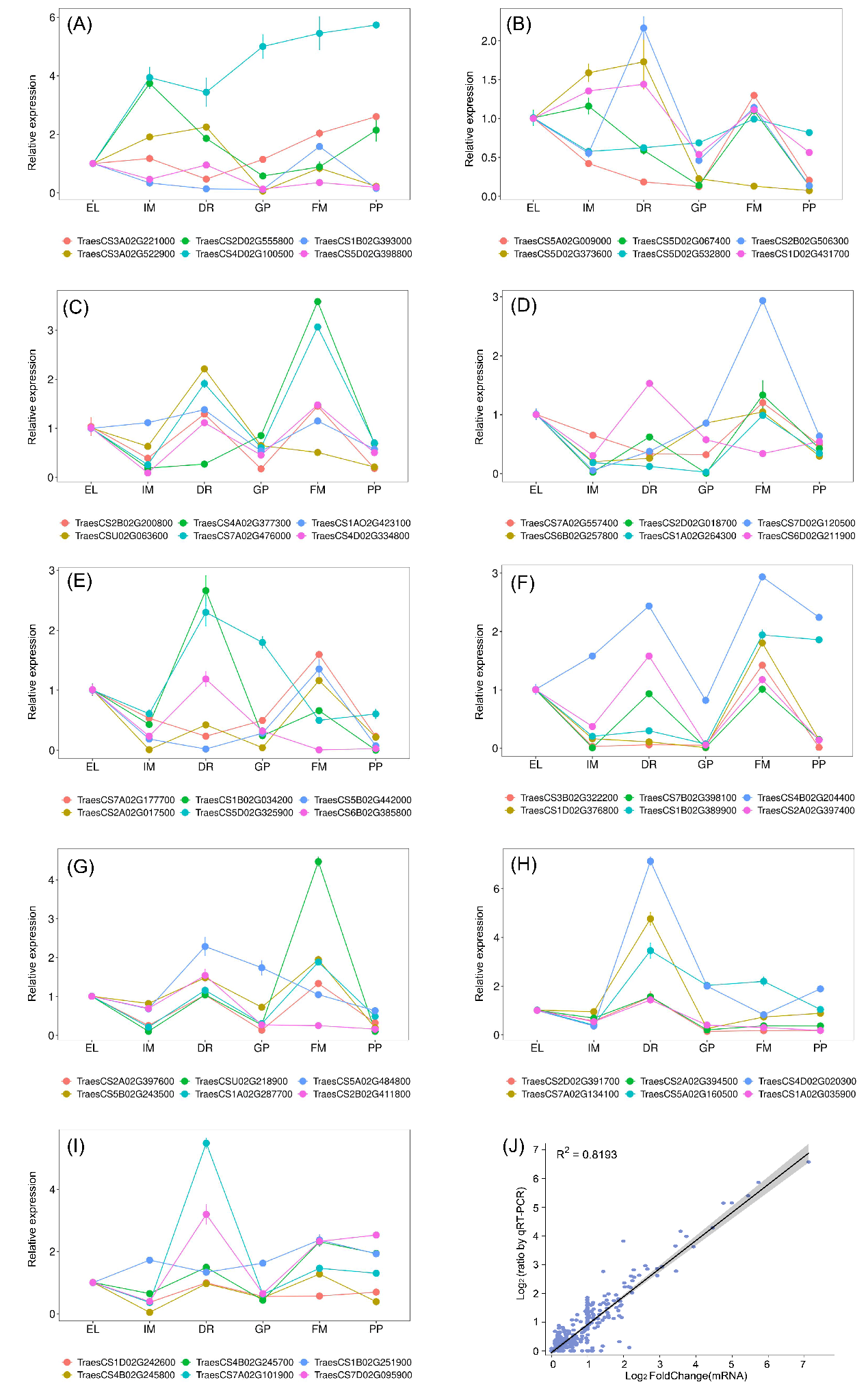


**Figure S7 qRT-PCR validation of transcriptional changes of 54 genes screened from DEGs**. (A−I) qRT-PCR validation of 54 genes normalized by β-actin gene; (J) transcriptional relationship between qRT-PCR and 54 expressed genes. The coefficient of determination (r^2^) is labeled in the figure. All qRT-PCR reactions were performed in three biological replicates.


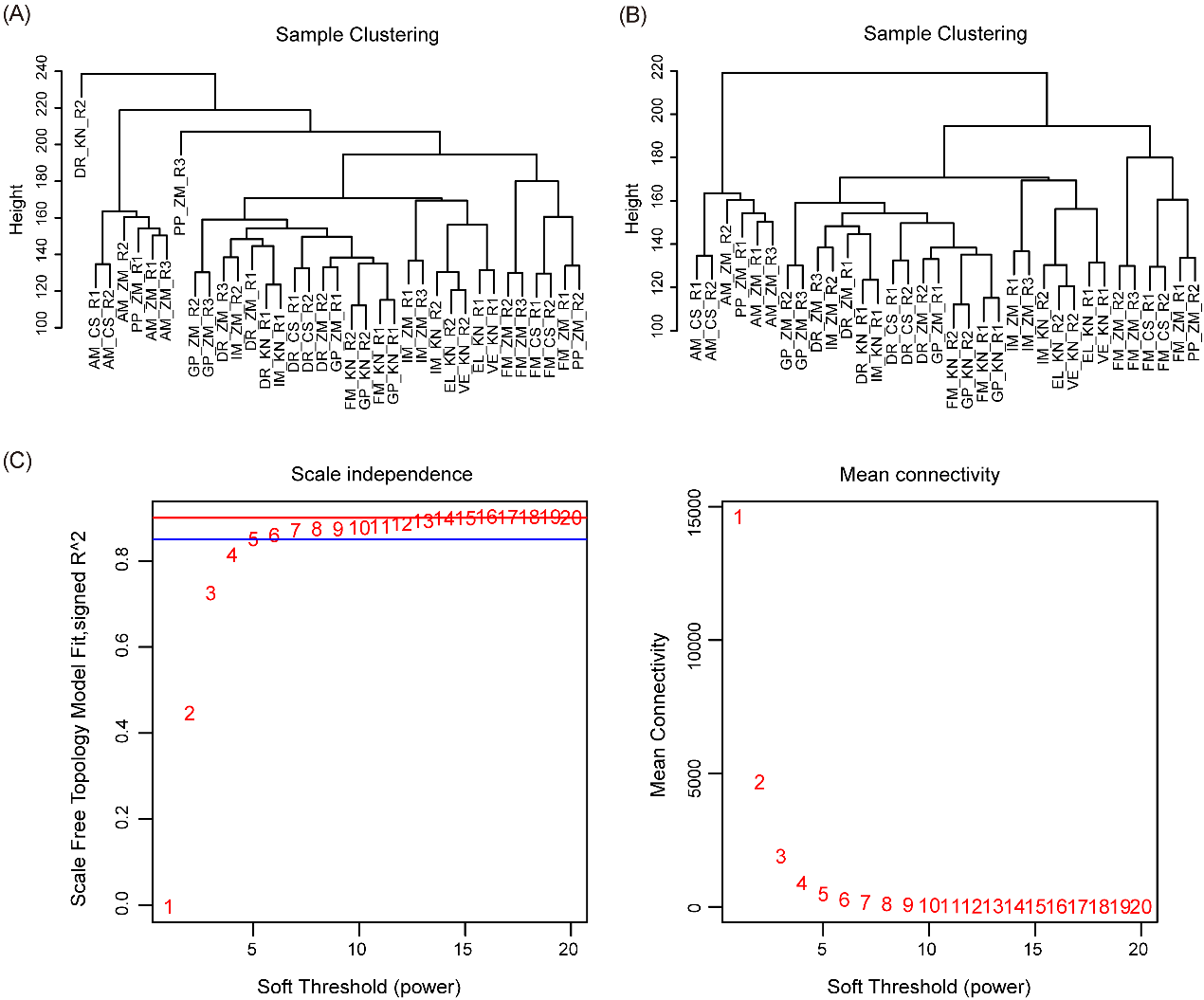


**Figure S8 Gene co-expression network.** (A) clustering tree of samples from the original data; (B) clustering tree after removing discrete samples; (C) selection of optimal soft threshold (power).


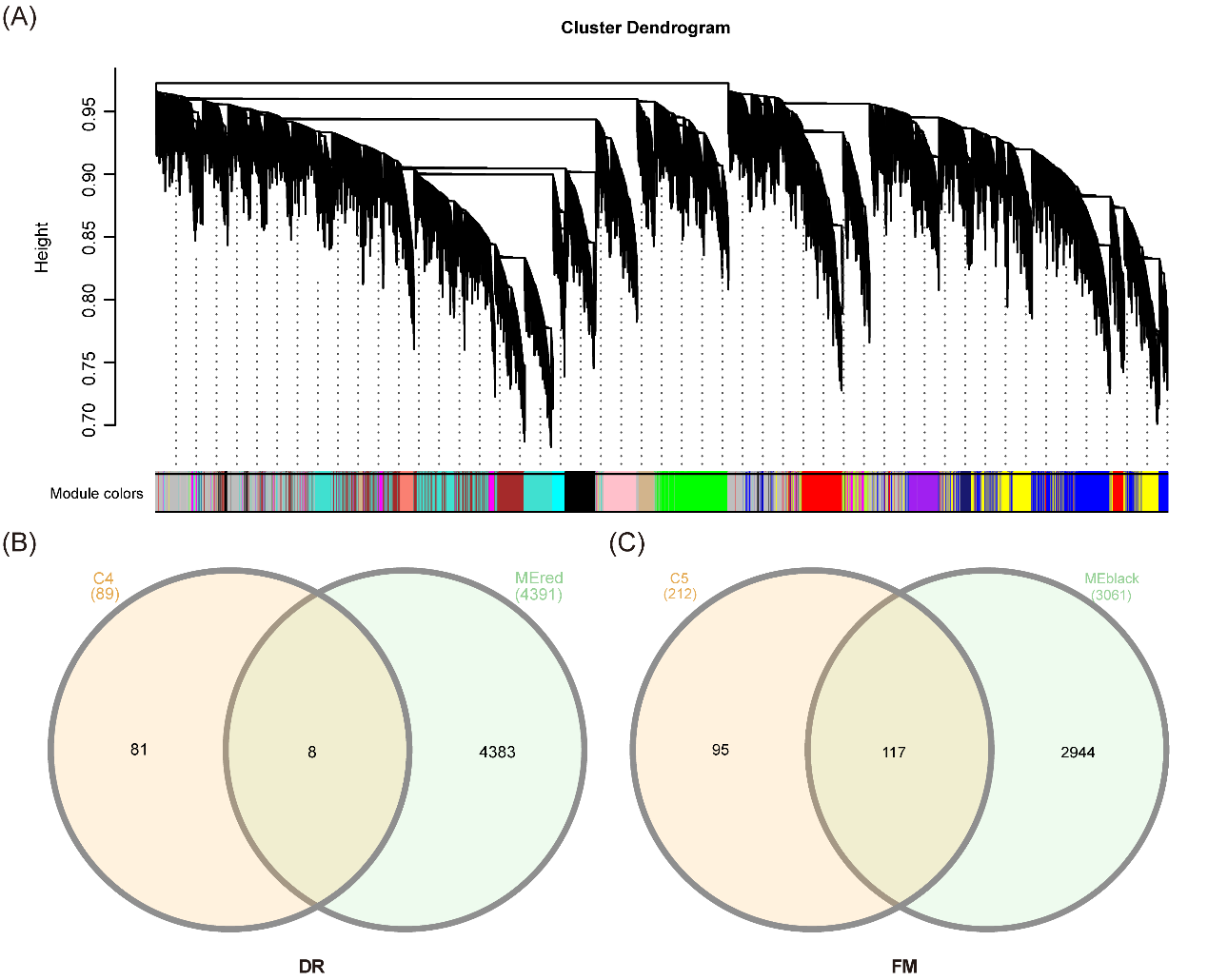


**Figure S9 Hub gene screening for wheat spike development**. (A) gene cluster tree and module construction; (B) venn plot of C4 and MEred module; (C) venn plot of C5 and MEblack module.


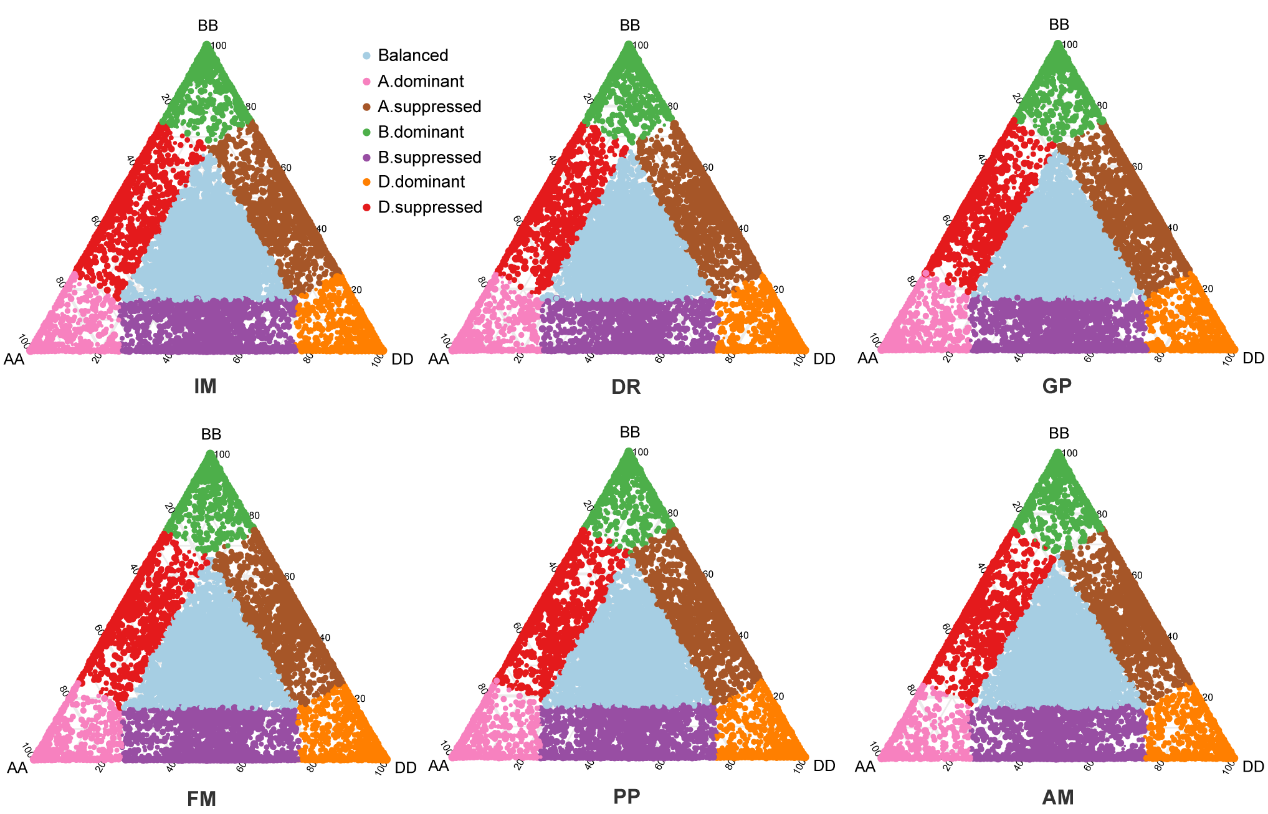


**Figure S10 Homeolog in different periods asymmetrical expression in syntenic homeologs triads.** Each circle represents a gene triad with an A, B, and D coordinate consisting of the relative contribution of each homeolog to the overall triad expression. Triads in vertices correspond to single-subgenome dominant categories, whereas triads close to edges and between vertices correspond to suppressed categories.


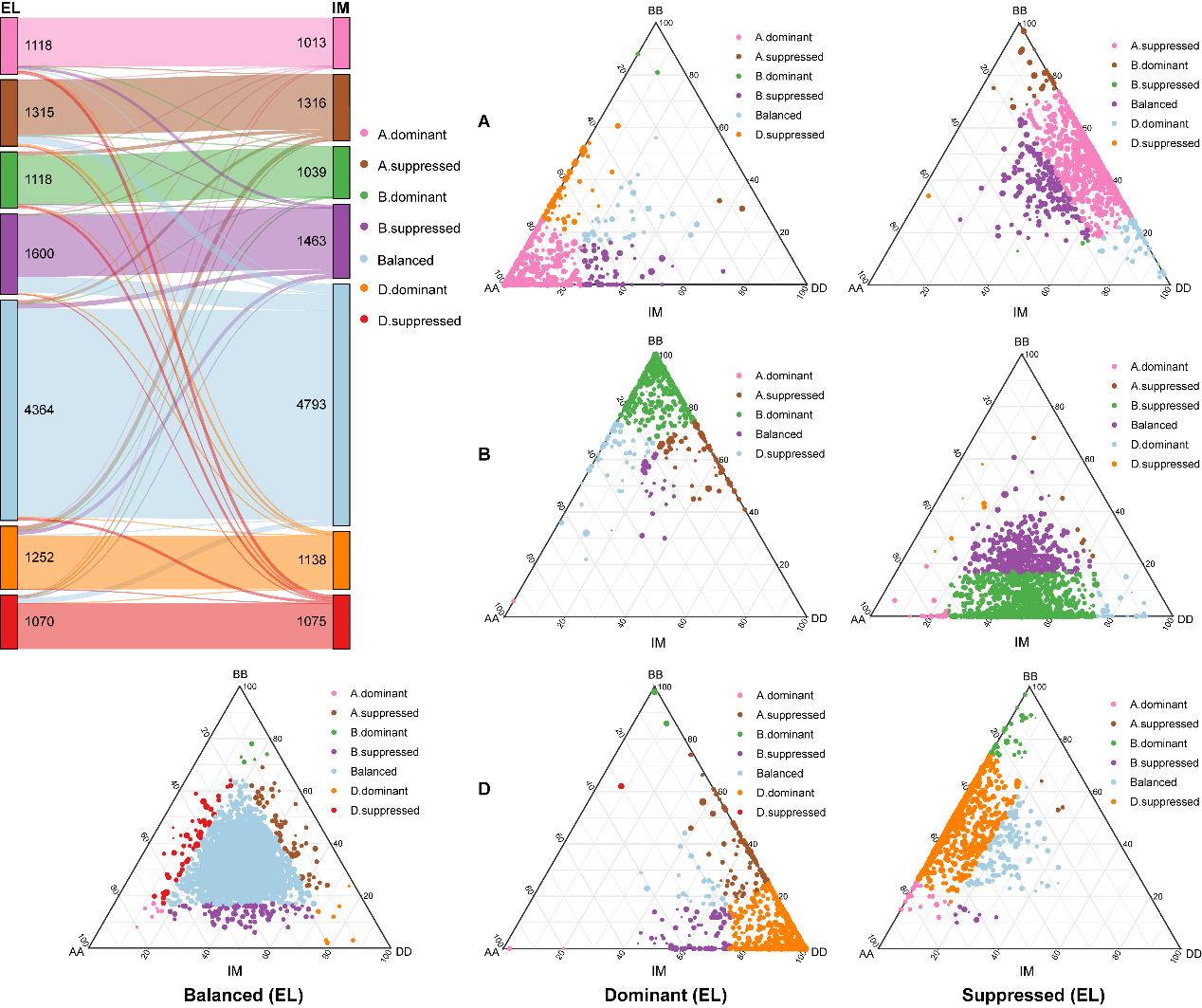


**Figure S11 Changes in the asymmetrical expression patterns from elongation stage to single ridge stage.**


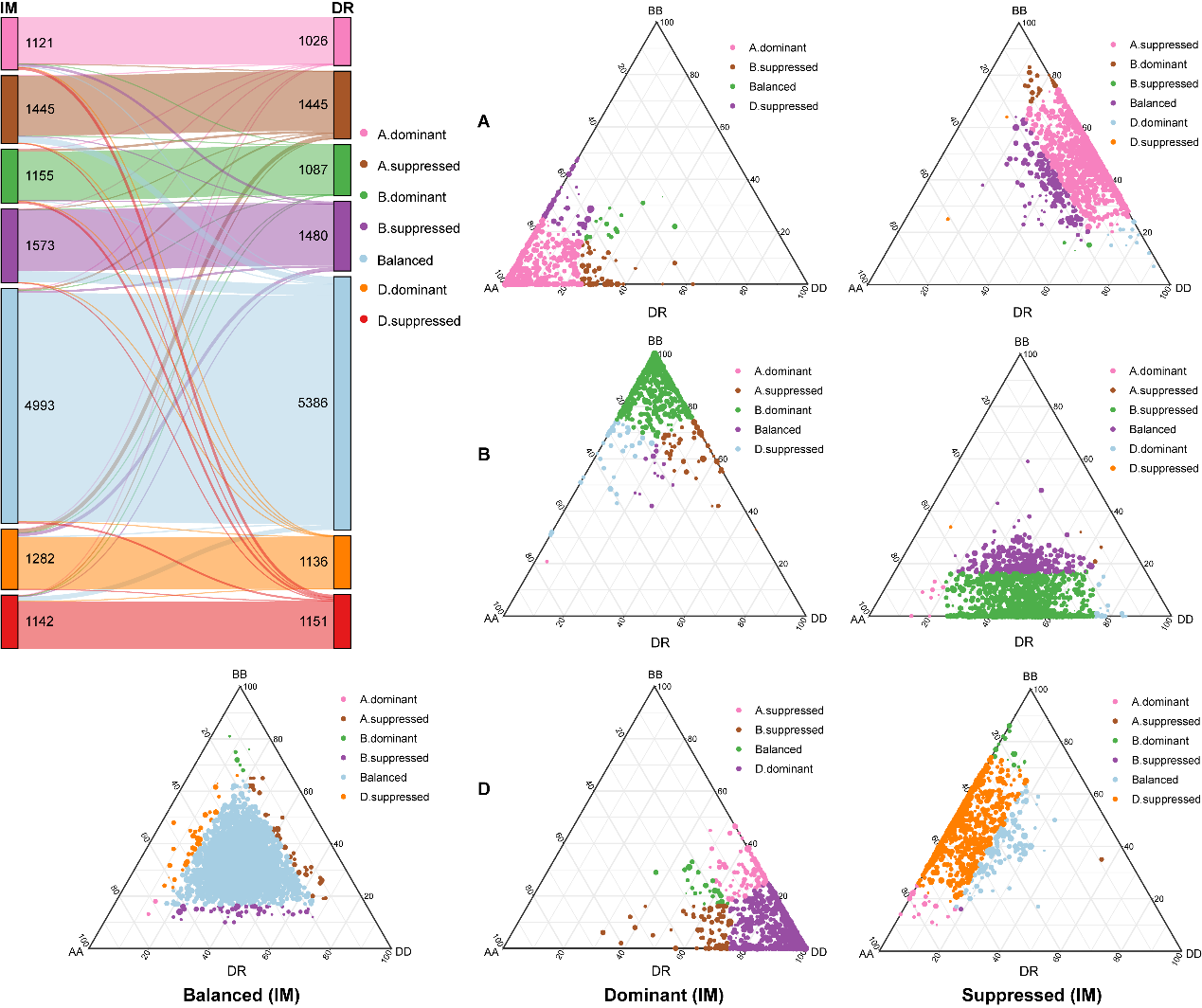


**Figure S12 Changes in the asymmetrical expression patterns from single ridge stage to double ridge stage.**


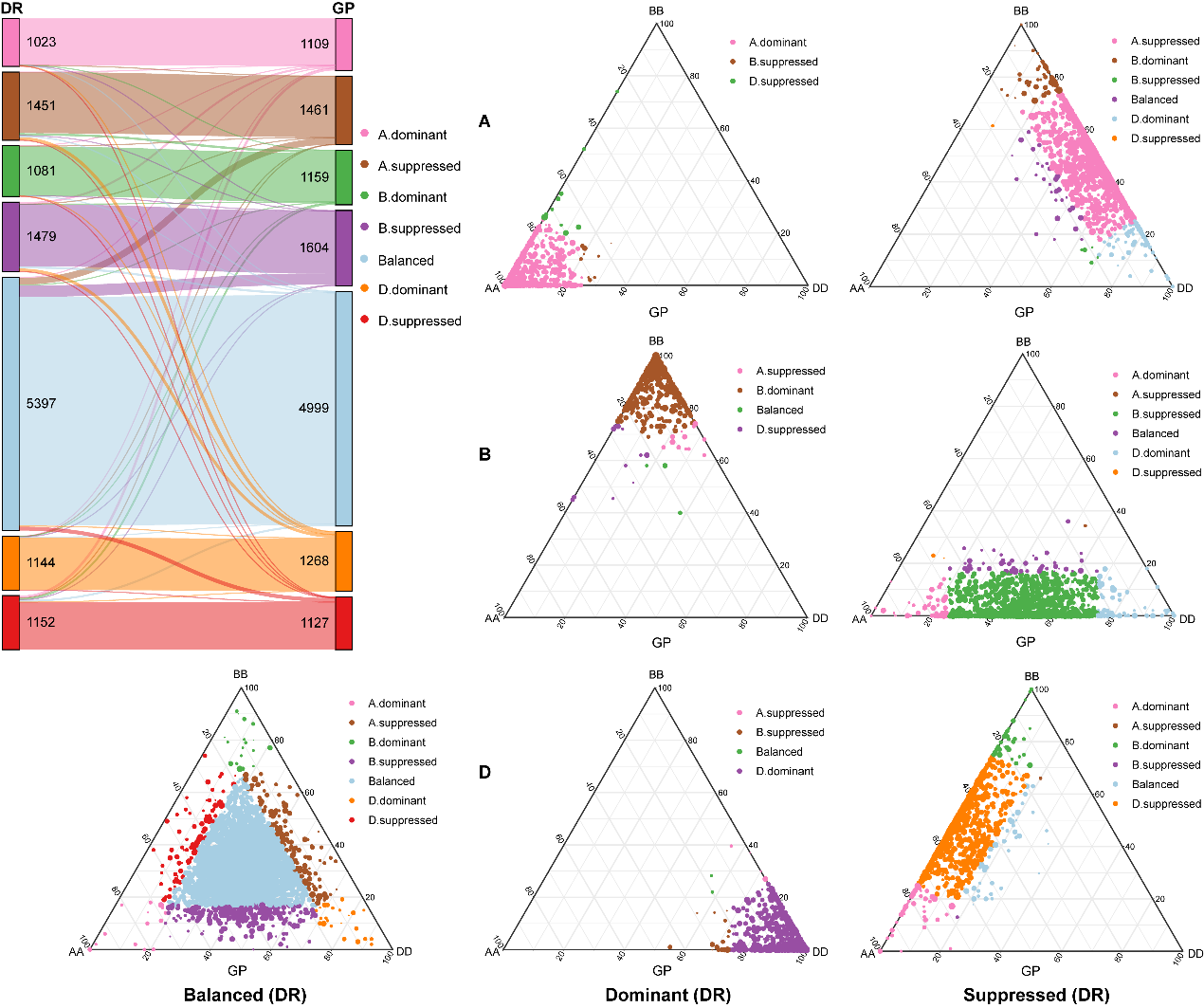


**Figure S13 Changes in the asymmetrical expression patterns from double ridge stage to glume primordium differentiation stage.**


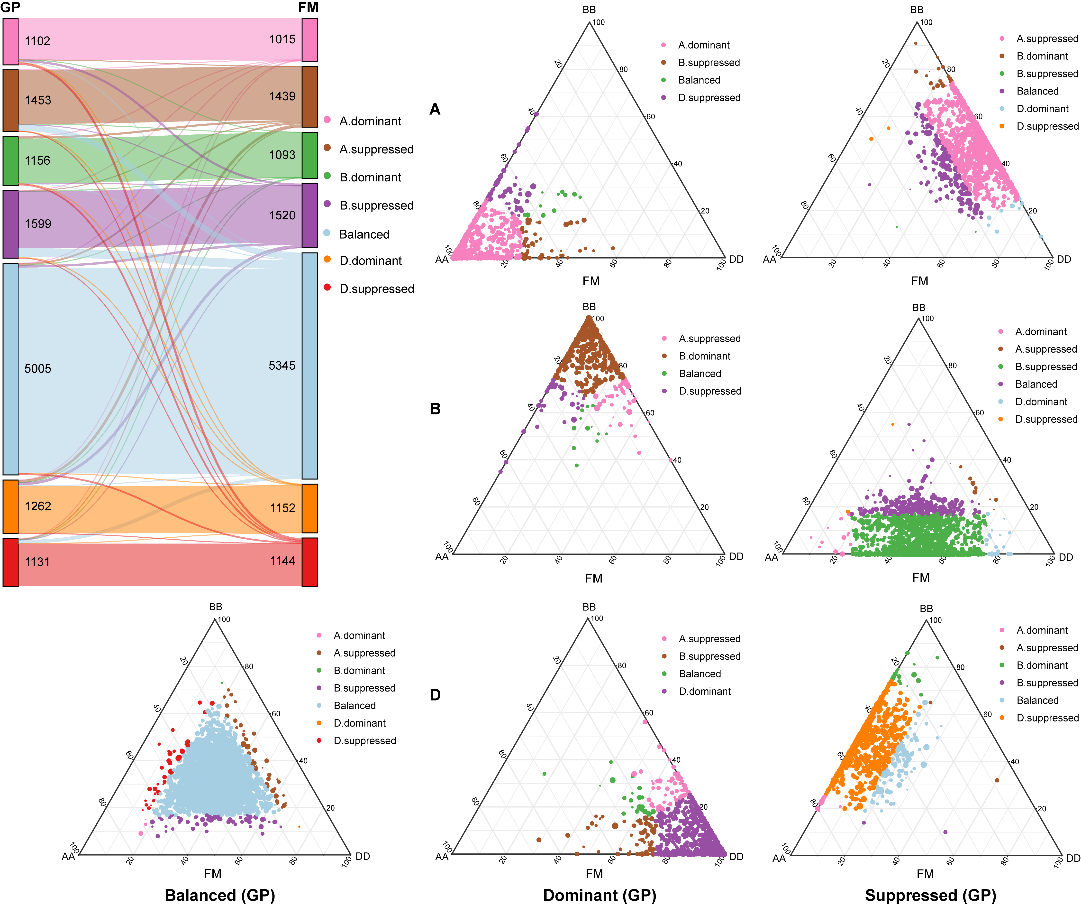


**Figure S14 Changes in the asymmetrical expression patterns from** **glume primordium differentiation stage to floral meristem stage.**


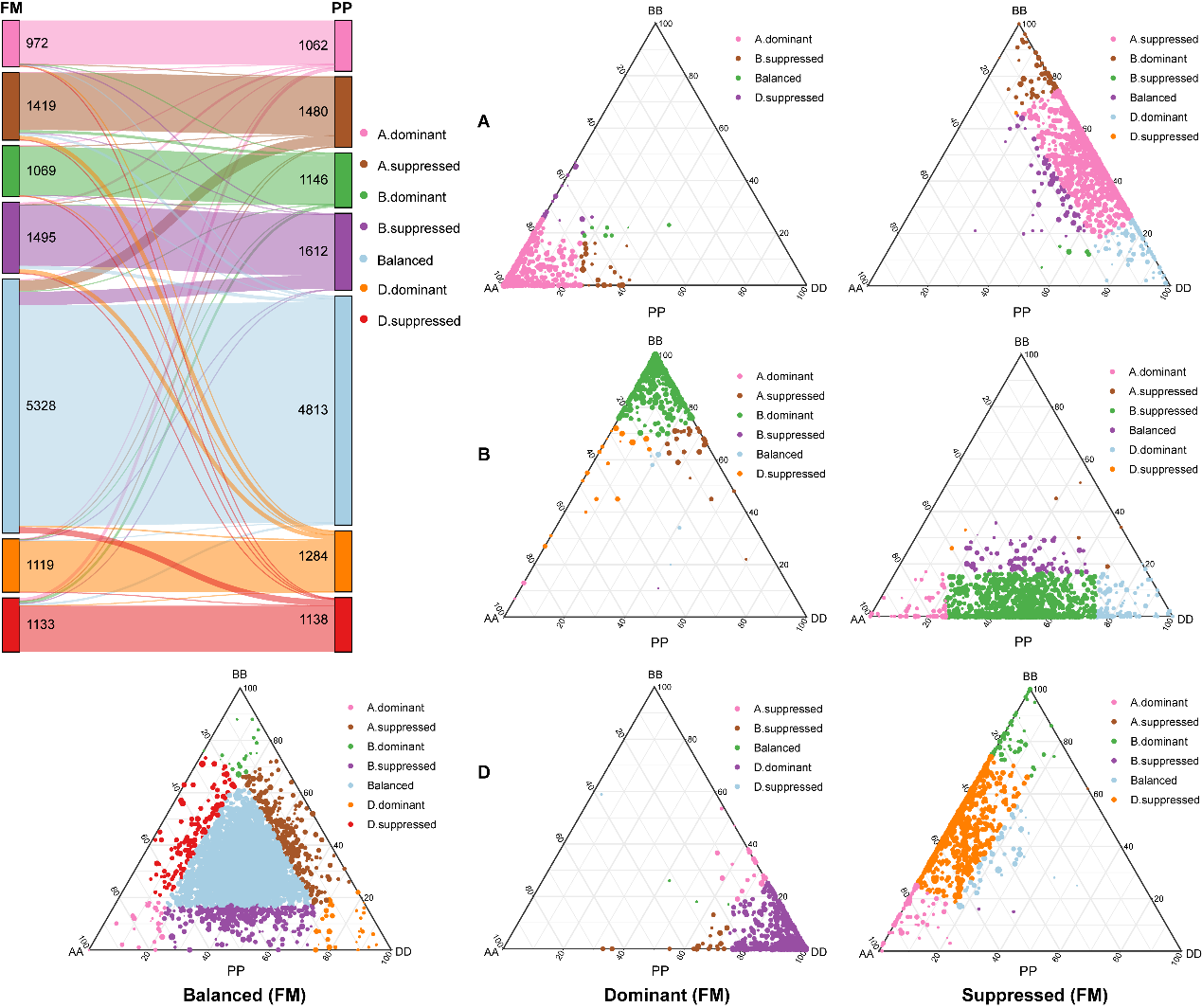


**Figure S15 Changes in the asymmetrical expression patterns from floral meristem stage to pistil primordium.**


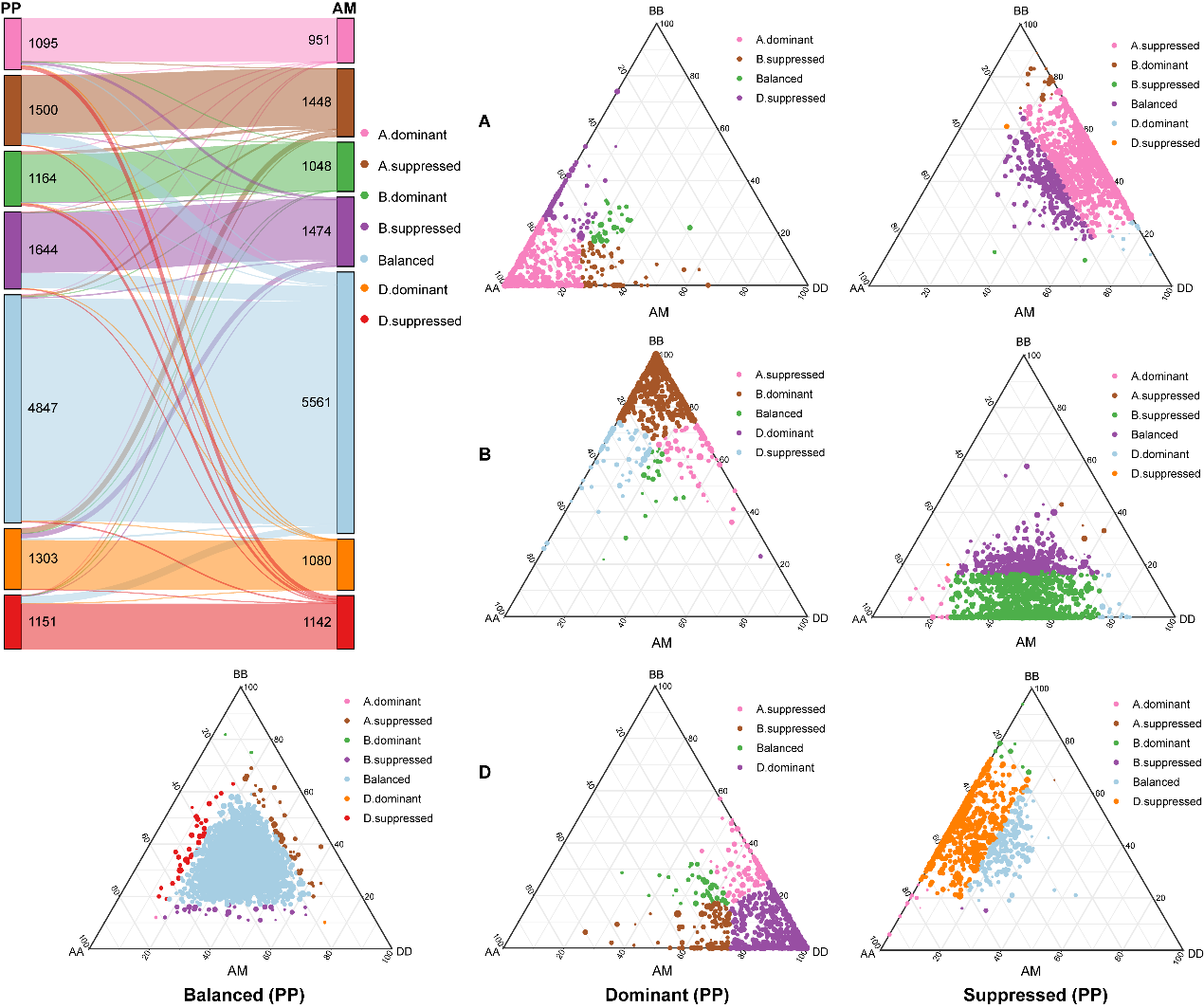


**Figure S16 Changes in the asymmetrical expression patterns from pistil primordium to anther meristem stage.**


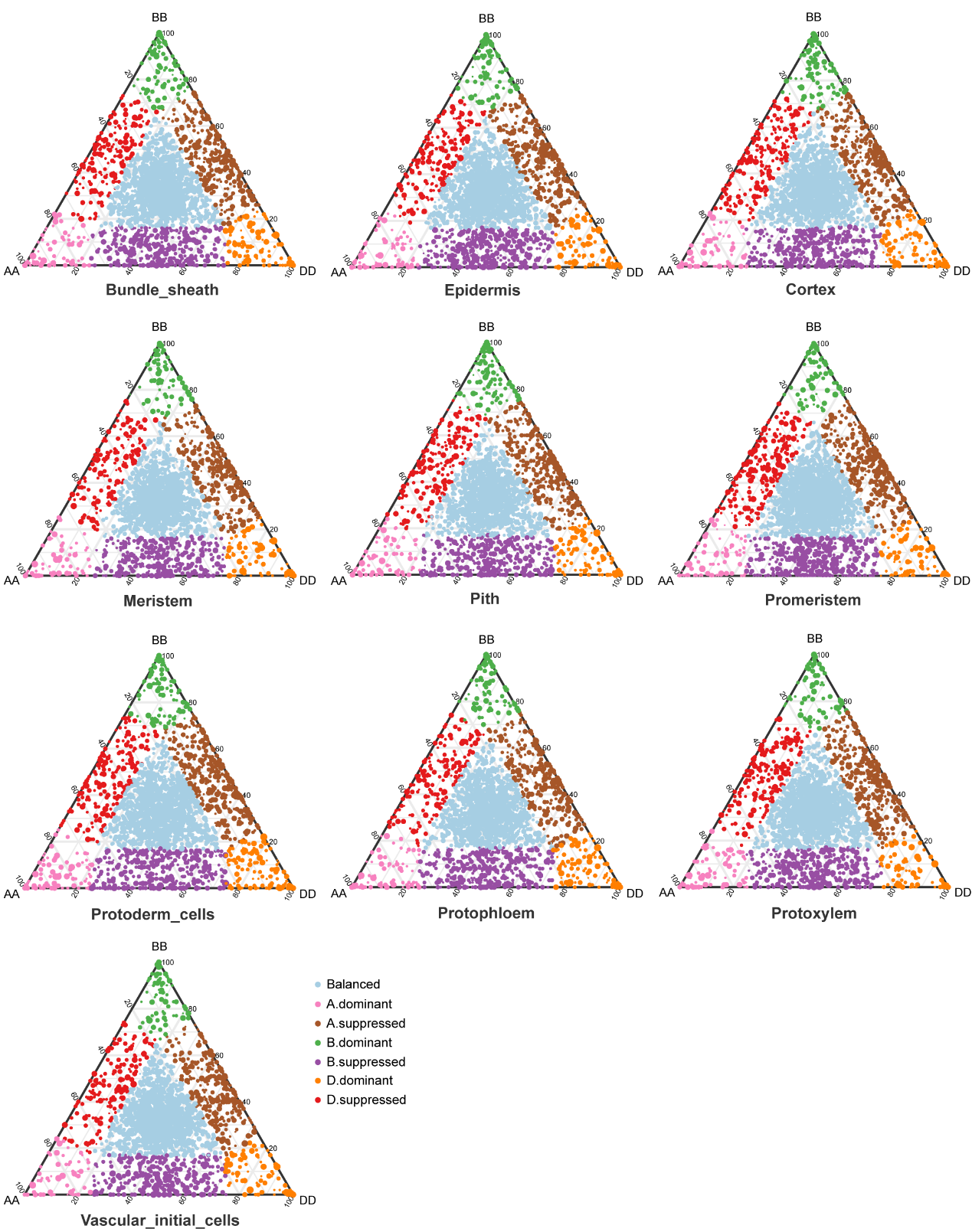


**Figure S17 Homeolog in different cell types asymmetrical expression in syntenic homeologs triads.** Each circle represents a gene triad with an A, B, and D coordinate consisting of the relative contribution of each homeolog to the overall triad expression. Triads in vertices correspond to single-subgenome dominant categories, whereas triads close to edges and between vertices correspond to suppressed categories.


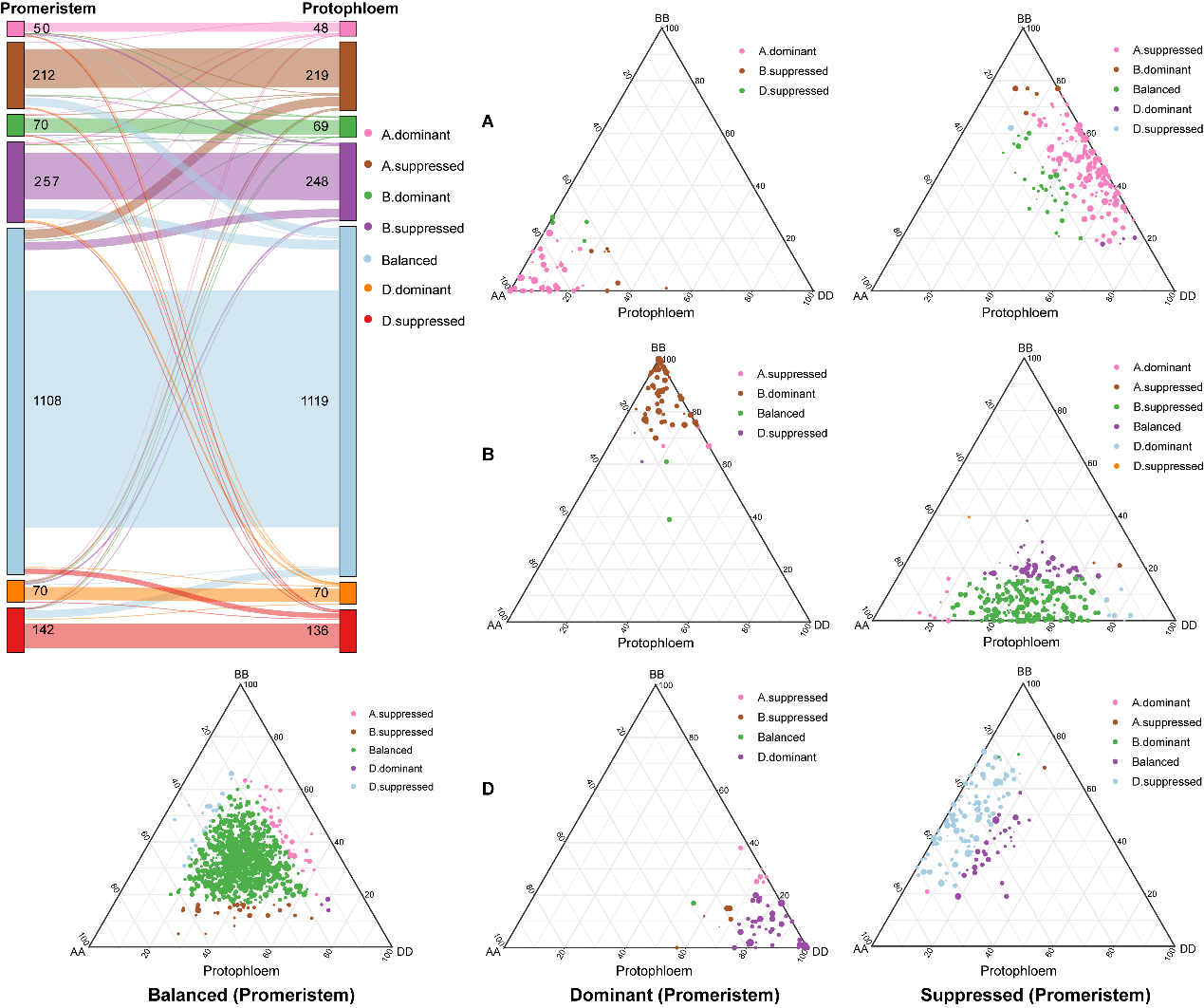


**Figure S18 Changes in the asymmetrical expression patterns from promeristem to protophloem.**


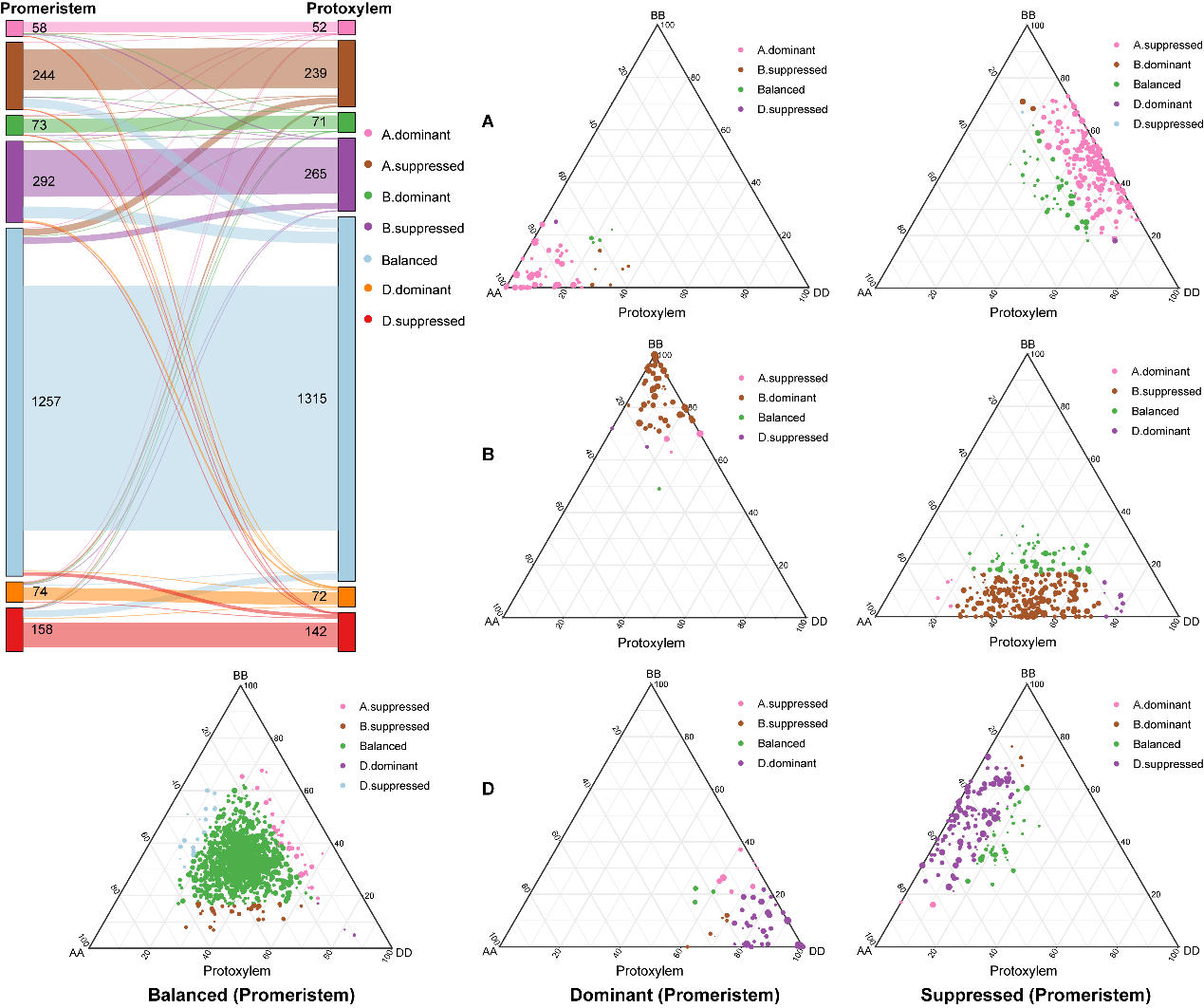


**Figure S19 Changes in the asymmetrical expression patterns from promeristem to** **protoxylem.**


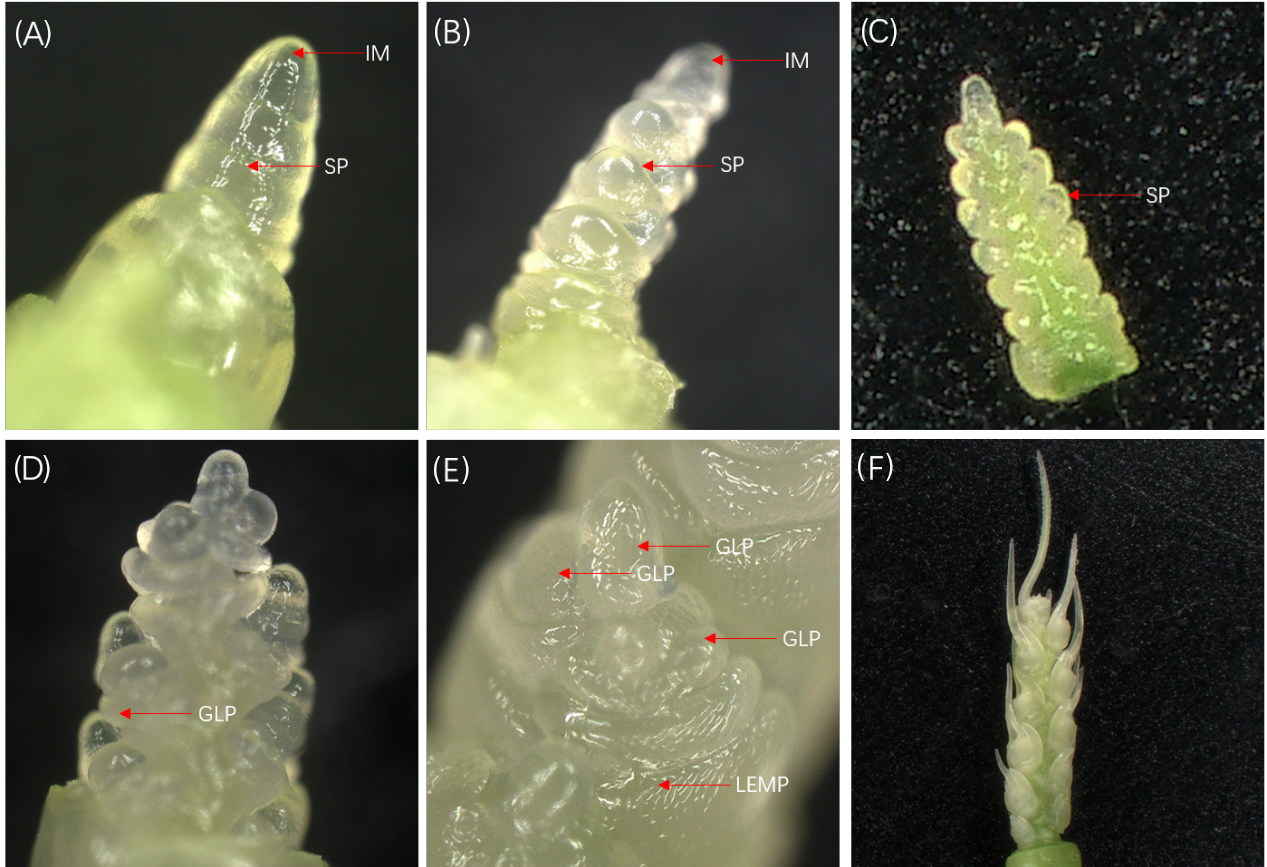


**Figure S20 Wheat spike at various developmental periods.** (A) single-ridge stage; (B) double-ridge stage; (C) glume primordium differentiation stage; (D) floral meristem stage; (E) local enlargement of floral meristem stage; (F) pistil primordium stage; SP: spike primordium; IM: apical growth center; GLP: guard glume primordium; LEMP: lemma primordium. Bars = 200 um
